# Enhanced Photodynamic Therapy Synergizing with Inhibition of Tumor Neutrophil Ferroptosis Boosts Anti‐PD‐1 Therapy of Gastric Cancer

**DOI:** 10.1002/advs.202307870

**Published:** 2024-01-17

**Authors:** Xudong Zhu, Wenxuan Zheng, Xingzhou Wang, Zhiyan Li, Xiaofei Shen, Qi Chen, Yanjun Lu, Kai Chen, Shichao Ai, Yun Zhu, Wenxian Guan, Shankun Yao, Song Liu

**Affiliations:** ^1^ Division of Gastric Surgery Department of General Surgery Nanjing Drum Tower Hospital Affiliated Hospital of Medical School Nanjing University Nanjing 210008 China; ^2^ China Pharmaceutical University Nanjing Drum Tower Hospital Nanjing 210008 China; ^3^ Department of Pharmacy Nanjing Drum Tower Hospital Affiliated Hospital of Medical School Nanjing University Nanjing 210008 China; ^4^ State Key Laboratory of Coordination Chemistry Coordination Chemistry Institute School of Chemistry and Chemical Engineering Nanjing University Nanjing 210023 China

**Keywords:** gastric cancer, ferroptosis, immunotherapy, neutrophils, photodynamic therapy

## Abstract

For tumor treatment, the ultimate goal in tumor therapy is to eliminate the primary tumor, manage potential metastases, and trigger an antitumor immune response, resulting in the complete clearance of all malignant cells. Tumor microenvironment (TME) refers to the local biological environment of solid tumors and has increasingly become an attractive target for cancer therapy. Neutrophils within TME of gastric cancer (GC) spontaneously undergo ferroptosis, and this process releases oxidized lipids that limit T cell activity. Enhanced photodynamic therapy (PDT) mediated by di‐iodinated IR780 (Icy7) significantly increases the production of reactive oxygen species (ROS). Meanwhile, neutrophil ferroptosis can be triggered by increased ROS generation in the TME. In this study, a liposome encapsulating both ferroptosis inhibitor Liproxstatin‐1 and modified photosensitizer Icy7, denoted LLI, significantly inhibits tumor growth of GC. LLI internalizes into MFC cells to generate ROS causing immunogenic cell death (ICD). Simultaneously, liposome‐deliver Liproxstatin‐1 effectively inhibits the ferroptosis of tumor neutrophils. LLI‐based immunogenic PDT and neutrophil‐targeting immunotherapy synergistically boost the anti‐PD‐1 treatment to elicit potent TME and systemic antitumor immune response with abscopal effects. In conclusion, LLI holds great potential for GC immunotherapy.

## Graphical abstract

Neutrophils are susceptible to ferroptosis and created an immunosuppressive tumor microenvironment in gastric cancer. The di‐iodinated photosensitizer IR780 (Icy7) is synthesized, ferroptosis inhibitor Liproxstatin‐1 and modified photosensitizer Icy7 are coloaded into liposomes (LLI) to induce ICD in tumor cells, inhibit neutrophil ferroptosis, remodel immunosuppressive TME, and work in synergy with anti‐PD‐1 antibody treatment.

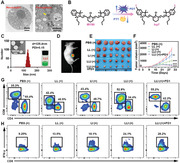

## 1 Introduction

Gastric cancer (GC) stands as the fifth most frequently diagnosed cancer, ranking as the fourth leading cause of all cancer‐related deaths (7.7%) in 2020.^[^ ^1^ ^]^ The incidence of gastric cancer is exceptionally high in Asia, particularly in countries like China, Korea, and Japan.^[^ ^2^ ^]^ For GC treatment, surgery remains the key curative approach. However, most cases are diagnosed at an advanced stage due to the late presentation of symptoms. Despite the advancements in surgical techniques, chemotherapy, and targeted therapy, the 5‐year overall survival rate of GC patients remains unsatisfactory.^[^ ^3^, ^4^, ^5^ ^]^ Hence, there is a pressing need for early diagnosis and the development of innovative anticancer therapies. The ultimate goal in tumor therapy is to eliminate the primary tumor, manage potential metastases, and trigger a systemic anti‐tumor immune response, ultimately leading to the clearance of all malignant cells.

Photodynamic therapy (PDT) is considered a promising strategy for cancer treatment duo to its precise temporal and spatial control, minimal invasiveness, and low side effects.^[^ ^6^, ^7^ ^]^ However, the commonly used photosensitizers are limited by their optimal excitation wavelength, falling within the UV‐visible range (200–700 nm),^[^ ^8^ ^]^ which restricts tissue penetration for in vivo application. To address this limitation, some strategies have been proposed by employing near‐infrared (NIR) light excitation for PDT.^[^ ^9^, ^10^, ^11^ ^]^ One such approach involves the use of IR780, a recently developed NIR heptamethine cyanine dye, employed for both PDT and photothermal therapy (PTT). IR780 has emerged as a promising nano‐agent for tumor therapies due to its higher optical stability and lower tissue autofluorescence compared to the widely used ICG (Indocyanine Green),^[^ ^12^ ^]^ and has therefore become a cancer imaging probe and a targeted therapeutic tool for cancer treatment.^[^ ^13^ ^]^ Upon activation by a specific wavelength of light, photosensitizers generate cytotoxic reactive oxygen species (ROS) that directly induce cell necrosis and/or apoptosis.^[^ ^14^ ^]^ In addition, immunogenic cell death (ICD) triggered by PDT also promotes the release of damage‐associated molecular patterns (DAMPs), including calreticulin (CRT), high mobility group box 1 (HMGB1), and adenosine triphosphate (ATP), effectively eliciting an antitumor immune response.^[^ ^15^, ^16^ ^]^ The effectiveness of PDT heavily relies on the intracellular production of ROS. However, achieving efficient PDT treatment has been a challenge duo to the extremely low quantum yield for singlet oxygen production in heptamethine cyanine dyes, including IR780.^[^ ^17^, ^18^ ^]^

Tumor microenvironment (TME) refers to the local biological environment of a solid tumor, consisting of tumor cells, non‐tumor cells, and extracellular matrix (ECM), and has increasingly become an attractive target for GC therapy.^[^ ^19^, ^20^ ^]^ A recent study has revealed a novel phenomenon where neutrophils within TME spontaneously undergo ferroptosis, and this process releases oxidized lipids that limit T cell activity.^[^ ^21^ ^]^ Notably, this finding contradicts the currently prevailing view of ferroptosis as a tumor‐growth‐limiting process or mechanism. Ferroptosis, introduced by Dixon et al. in 2012, refers to a newly identified mechanism of regulated cell death (RCD) mediated by iron‐dependent lipid peroxidation of cell membranes, characterized by unique morphological, biochemical, and genetic features.^[^ ^22^ ^]^ Besides, ferroptosis can be triggered either by increasing cellular iron load and ROS generation or by inhibiting the antioxidant machinery.^[^ ^23^, ^24^ ^]^ Although ferroptosis inducers are highly effective in killing tumor cells in vitro, they have shown little effect in experimental animal models in vivo, except in immunodeficient mice.^[^ ^21^ ^]^ This indicates the incomplete understanding of the role of ferroptosis in immune cells and the poorly elucidated types and impact of ferroptosis within the TME of gastric cancer.

**Scheme 1:**
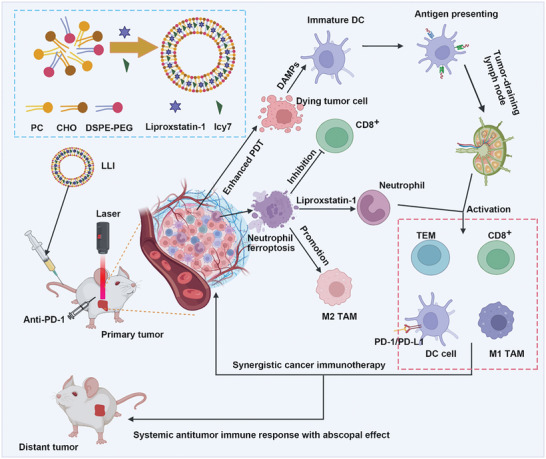
Schematic illustration of the preparation of multifunctional nanodrug utilizing liposome to co‐encapsulate ferroptosis inhibitor Liproxstatin‐1 and modified photosensitizer Icy7 (denoted LLI). LLI‐based immunogenic PDT and neutrophil‐targeting immunotherapy synergistically boosted the anti‐PD‐1 therapy to elicit potent TME and systemic antitumor immunity with abscopal effects.

Current immunotherapy treatments, especially immune checkpoint blockages (ICBs), have demonstrated enhanced outcomes in an increasing range of cancer types.^[^ ^25^, ^26^ ^]^ Nevertheless, a substantial proportion of cancer patients remain unresponsive to existing ICBs treatments. This is partly due to immunosuppressive TME or a lack of pre‐existing antitumor immune cells.^[^ ^27^, ^28^ ^]^ Multiple strategies have been studied to address these challenges, encompassing approaches such as cancer vaccines, chemotherapy, radiotherapy, PDT, and oncolytic virotherapy.^[^ ^29^, ^30^, ^31^, ^32^ ^]^ Clinical and experimental data have revealed the upregulation of programmed death ‐ligand 1 (PD‐L1) expression in response to various anti‐tumor therapies, such as PDT, chemotherapy, and radiotherapy.^[^ ^33^, ^34^, ^35^ ^]^ Significantly, PDT demonstrated its efficacy in treating cancer, making it a suitable candidate for combination treatment with immunotherapy, offering both an immediate and sustained antitumor response. In addition to this, the inhibition of neutrophil ferroptosis has been shown to restore the function of T cells by decreasing the level of oxidized lipids, thus further enhancing its synergy with ICBs in suppressing tumor growth.^[^ ^21^ ^]^ This study aims to investigate ferroptosis in various cell types within TME and explore its role in the pathophysiology of GC. Subsequently, we developed an immunoregulatory nanodrug by employing liposome to co‐encapsulate the ICD‐inducer Icy7 and ferroptosis inhibitor Liproxstatin‐1 (LLI). The nanoscale LLI was designed to accumulate in GC tissues, thereby inducing ICD of tumor cells and inhibiting ferroptosis of neutrophils within TME to reverse the immunosuppressive microenvironment, thereby boosting the immunotherapy of GC (**Scheme** **1**).

## 2 Results

### 2.1 Tumor Neutrophils were Susceptible to Ferroptosis

**Figure 1:**
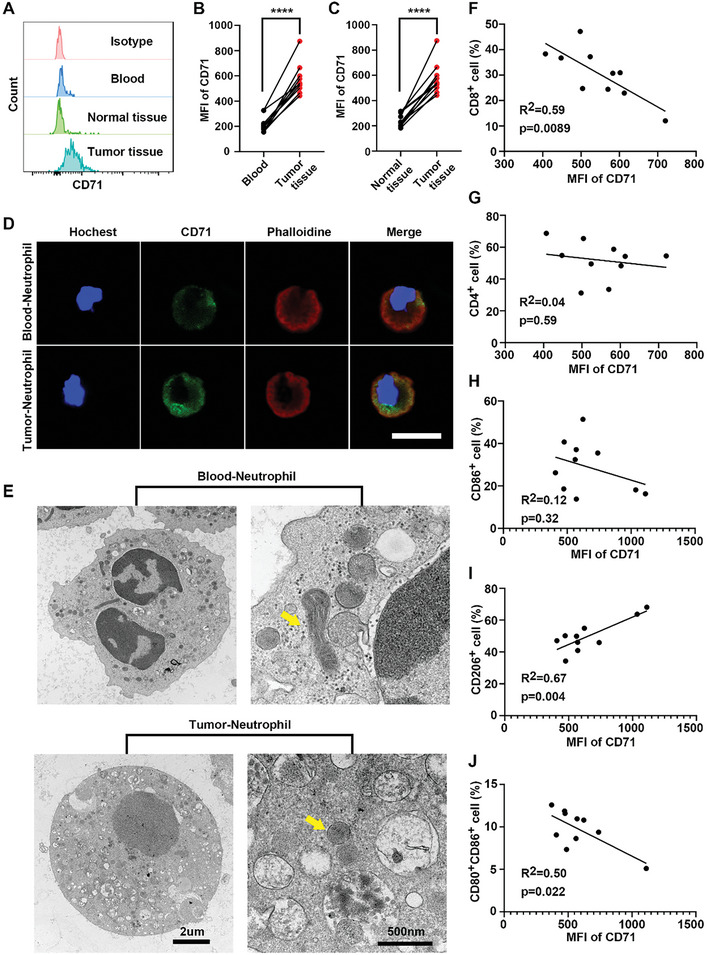
Intra‐tumoral neutrophils were susceptible to ferroptosis and created an immunosuppressive tumor microenvironment. A) Representative FACS images showed the ferroptosis of neutrophils from the paired peripheral blood, normal tissue, and tumor tissue of patients with gastric cancer. B,C) CD71 was measured and analyzed, *n* = 10. D) Immunofluorescence analysis of neutrophils (hochest; blue), (CD71; green), and (phalloidine; red) in gastric cancer samples. Scale bar, 25 µm. E) Transmission electron microscope images of neutrophils in gastric cancer samples. Scale bars, 2 µm, and 500 nm. (F and G) Correlation analysis of the MFI of CD71 with CD8^+^ and CD4^+^ T cells, *n* = 10. (H and I) Correlation analysis of the MFI of CD71 with the M1 and M2 TAMs, *n* = 10. (J) Correlation analysis of the MFI of CD71 with the maturation of DC, *n* = 10. (Data were presented as the mean ± SD; ^****^ *p* < 0.0001).

Ferroptosis in neutrophils from paired peripheral blood, normal tissues, and tumor tissues of 10 patients with GC was determined by flow cytometry. Gating strategies were used to investigate the proportion of immune cells in human GC samples (Figure S1, Supporting Information). The results showed higher levels of CD71, a known cellular marker of ferroptosis, in intra‐tumoral neutrophils, indicating their susceptibility to ferroptosis (**Figure** **1**A–C). Higher CD71 levels were also shown by immunofluorescence staining of neutrophils from GC tissues compared with neutrophils from paired peripheral blood samples (Figure 1D). Morphologically, ferroptosis was associated with shrunken mitochondria with loss of cristae, cytoplasmic swelling, and cell membrane rupture (Figure 1E). CD8^+^ T cells are the most powerful effector cells in the anti‐tumor immune response.^[^ ^36^ ^]^ The neutrophil ferroptosis was found to be negatively correlated with CD8^+^ but not CD4^+^ T cells (Figure 1F,G). Furthermore, neutrophil ferroptosis was positively correlated with the levels of CD206^+^ macrophages (M2 TAMs), while showing no significant correlation with CD86^+^ macrophages (M1 TAMs) (Figure 1H,I). Meanwhile, neutrophil ferroptosis was negatively correlated with the maturation of dendritic cells (DCs) (Figure 1J). These results indicated that neutrophil ferroptosis created an immunosuppressive TME in GC. More importantly, it was the neutrophils (CD11b^+^CD15^+^ cells) in the TME that expressed more CD71 (Figure S2, Supporting Information), suggesting neutrophil ferroptosis as a potential therapeutic target for GC.

### 2.2 Preparation and Characterization of Nanodrugs

**Figure 2:**
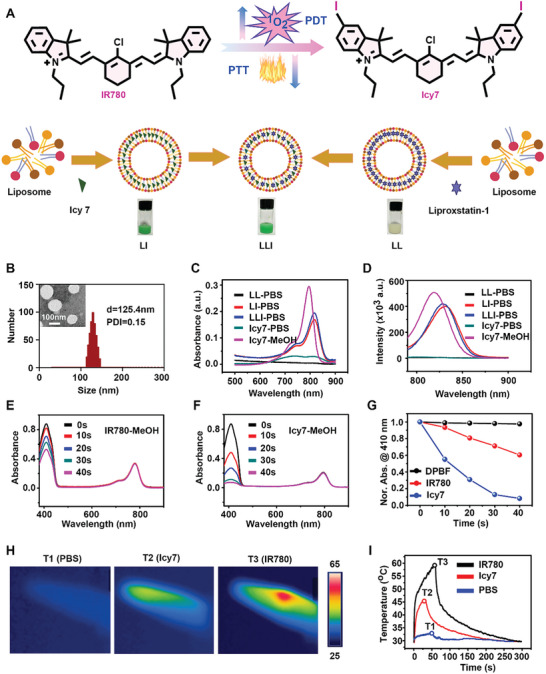
Characterization of nanodrugs. A) Chemical structures of IR780 and Icy7, and photographs of nanodrugs. B) Size distribution and transmission electron microscope image of LLI. C) Absorption spectra of LL, LI, LLI, and free Icy7 in PBS, and free Icy7 in MeOH. D) Fluorescence spectrum of LL, LI, LLI, and free Icy7 in PBS, and free Icy7 in MeOH. (E–G) Absorption spectra of 1,3‐diphenylisobenzofuran (DPBF), and degradation of DPBF absorption corresponds to the production of ^1^O_2_ induced by IR780 and Icy7 in MeOH under 808 nm light irradiation (0.5 W cm^−2^). (H,I) Thermal images of IR780 and Icy7 solution in PBS recorded by FLIR thermal mapping camera under 808 nm light irradiation.

Lecithin (PC), cholesterol (CHO), and 1,2‐distearoyl‐sn‐glycero‐3‐phosphoe‐Thanolamine‐N‐[methoxy (polyethylene glycol)−2000] (DSPE‐PEG2000) were used for liposome formation. The di‐iodinated photosensitizer IR780 (Icy7) was synthesized, and the successful synthesis was confirmed by ^1^H NMR, ^13^C NMR, and HR‐MS spectra (Supporting Information). Heavy atoms were incorporated into the IR780, and these induced a greater generation of ROS with a decreased PTT effect (**Figure** **2**A). The ferroptosis inhibitor Liproxstatin‐1 and modified photosensitizer Icy7 were co‐loaded into liposomes to yield the nanodrug (LLI), as described in the methods section. Liproxstatin‐1‐encapsulated and Icy7‐encapsulated liposomes (LL and LI, respectively) were prepared for control experiments. As measured by dynamic light scattering (DLS), LLI showed an average diameter of 124.8 ± 1.3 nm, and exhibited spherical morphology under transmission electron microscopy (TEM) (Figure 2B). The size, polydispersity (PDI), and Zeta potential of blank liposome, LL, and LI were similar to that of LLI (Figure S3, Supporting Information). The encapsulation efficiency of Icy7 and Liproxstatin‐1 was 61.1% and 58.0% in LLI, respectively. The addition of DSPE‐PEG2000 improved the stealth properties of the liposome (circulation time, stability, and targeting capability).^[^ ^37^ ^]^ The stability of LLI was measured in PBS containing 10% FBS, with no obvious change in size observed within 72 h (Figure S4A, Supporting Information). Following Icy7 encapsulation, LI and LLI showed absorption peaks at 816 nm in PBS, and exhibited a slight red‐shift from 795 nm of free Icy7, LI, and LLI in MeOH (Figure 2C; Figure S4B, Supporting Information). LI and LLI showed similar fluorescence intensities in PBS, and a visible decrease in the fluorescence intensity of free Icy7 was observed in PBS (Figure 2D). Moreover, compared with IR780, Icy7 maintained the photosensitivity with higher ^1^O_2_ generation in MeOH and PBS, relating to the presence of the heavy atoms in Icy7 (Figure 2E–G; Figure S5, Supporting Information). Under 808 nm laser irradiation (0.5 W cm^−2^), the temperature of the IR780 solution increased markedly, reaching a maximum of 59.1 °C. Similarly, the temperature of the Icy7 solution increased slightly, to a final temperature of 45.6 °C (Figure 2H,I). The incorporation of heavy atoms in IR780 caused greater ROS generation with a decreased PTT effect which may be due to the balance of the energy distribution.

### 2.3 In Vitro Cytotoxicity

**Figure 3:**
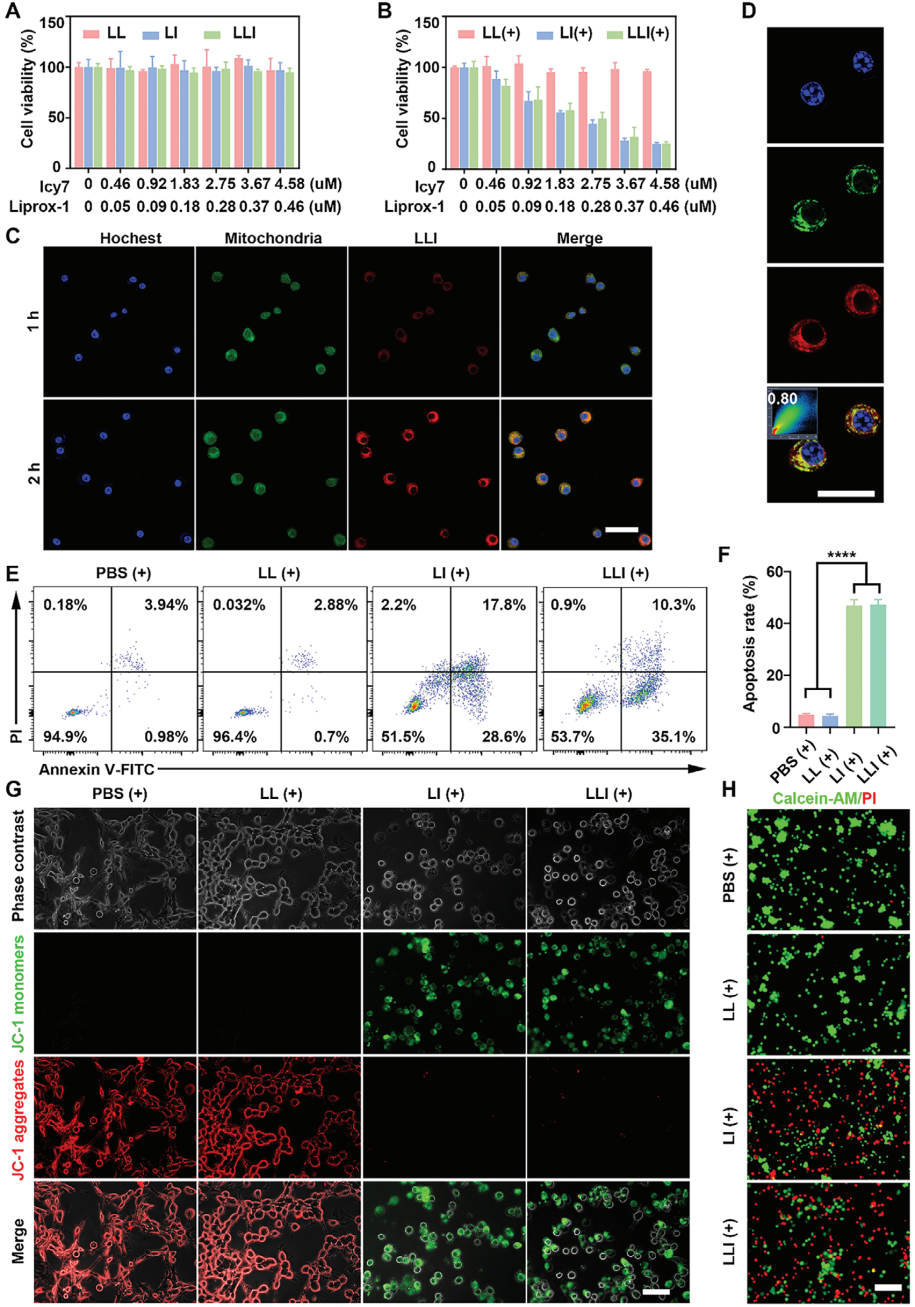
Cytotoxicity of nanodrugs on MFC cells in vitro. A,B) Cytotoxicity of LL, LI, and LLI without or with light irradiation in vitro (808 nm, 0.5 W/cm^2^, 1 min) (*n* = 3). C) LLI was internalized into MFC cells (hochest; blue), (mitochondria; green), and (LLI; red). Scale bar, 50 µm. D) colocalization of LLI with mitochondria. Scale bar, 25 µm. (E and F) The apoptosis rate of MFC cells was determined by flow cytometry in different groups, *n* = 3. G) Mitochondria membrane potential of MFC cells in different groups (JC‐1 monomers; green), (JC‐1 aggregates; red), *n* = 3. Scale bar, 50 µm. H) Live/dead cell staining of MFC cells in different groups (liver cells; green), and (dead cells; red), *n* = 3. Scale bar, 100 µm. (Data were presented as the mean ± SD; ^****^ *p* < 0.0001).

To assess the cytotoxicity of the nanodrugs, MFC cells were incubated with different concentrations of LL, LI, and LLI with or without laser exposure. There were no obvious changes in cell viability at the different nanodrug concentrations in the absence of irradiation (**Figure** **3**A). However, with laser irradiation, both LI and LLI showed significant concentration‐dependent cytotoxicity. The 50% inhibitory concentration (IC50) was ≈2.5 µM of Icy7 for both LI and LLI, indicating that cancer cells were killed by ROS generated by the endocytosed photosensitizer under appropriate laser irradiation (Figure 3B). The evaluation of nanodrug uptake by MFC cells showed significantly increased uptake at 2 h relative to that at 1 h (Figure 3C). Additionally, colocalization experiments showed good co‐localization of LLI with the mitochondria (Pearson's coefficient 0.80) in MFC cells (Figure 3D). The red fluorescence of DiI begins to fade after 12 h, indicating the release of nanodrugs after coculturing with cells (Figure S4C, Supporting Information). Measurement of the effects of the nanodrug on MFC apoptosis indicated apoptosis rates of 3.71%, 3.34%, 4.48%, and 4.62% in the PBS, LL, LI, and LLI groups, respectively, in the absence of laser irradiation (Figure S6A,B, Supporting Information). After irradiation, the apoptosis rates of MFC cells were 4.92, 3.58, 46.4, and 45.4% in the PBS (+), LL (+), LI (+), and LLI (+) groups, respectively (Figure 3E,F). It has been reported that 3D spheroids can better mimic the TME.^[^ ^38^ ^]^ The cell uptake and apoptosis with the treatment of different nanodrugs were also assessed in the 3D tumor spheroid models, and the results were consistent with the previous experiments (Figure S6C–G, Supporting Information). In addition, changes in mitochondrial membrane potentials were assessed using a JC‐1 kit. Normal cells with a higher mitochondrial membrane potential were observed as the dominant population of red fluorescence (JC‐1 aggregates). Whereas, the LI (+) and LLI (+) groups exhibited marked decreases in mitochondrial membrane potential with most cells emitting green fluorescence (JC‐1 monomers) (Figure 3G; Figure S7, Supporting Information). Consistently, live/dead cell staining confirmed increases in the numbers of dead cells (red fluorescence) in the LI (+) and LLI (+) groups compared to the LL (+) and PBS (+) groups following irradiation (Figure 3H), while the proportions of live and dead cells were similar in the different groups in the absence of laser irradiation (Figure S8, Supporting Information).

### 2.4 PDT‐Elicited ICD in Vitro

**Figure 4:**
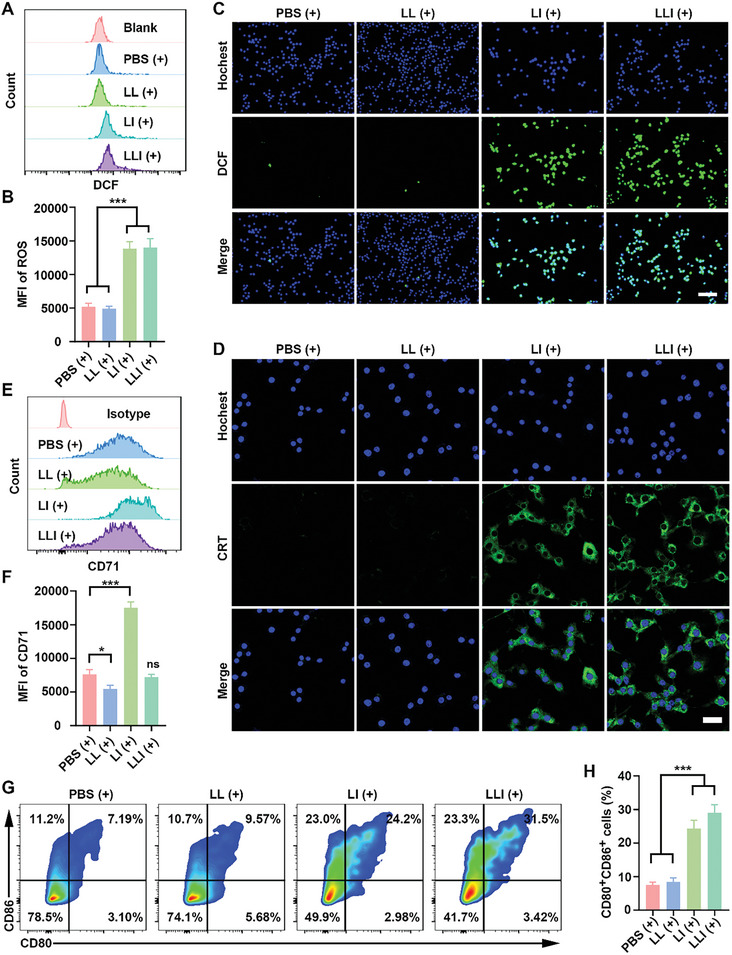
PDT‐mediated immunogenic cell death of MFC cells in vitro. A,B) ROS production in MFC cells after various treatments detected by flow cytometry, *n* = 3. (C) Fluorescence imaging of ROS production in MFC cells after various treatments (hochest; blue), and (DCF; green), *n* = 3. Scale bar, 100 µm. (D) Fluorescence imaging of CRT in MFC cells after various treatments (hochest; blue), and (CRT; green), *n* = 3. Scale bar, 25 µm. (E and F) CD71 expression of MFC cells detected by flow cytometry after various treatments, *n* = 3. (G and H) DCs maturation was determined by flow cytometry after being cocultured with nanodrugs‐pretreated MFC cells, *n* = 3. (Data were presented as the mean ± SD, ^*^ *p* < 0.05, ^***^ *p* < 0.001, and ns, not significant).

PDT effectively triggered immunogenic cell death (ICD), and promoted the release of key DAMPs associated with ICD, including CRT, HMGB1, and ATP.^[^ ^39^ ^]^ ROS generation was investigated using flow cytometry and CLSM in MFC cells treated with the different nanodrugs. The mean fluorescence intensity (MFI) of ROS in MFC cells was markedly increased in the LI (+) and LLI (+) groups compared to the LL (+) and PBS (+) groups, as shown by flow cytometry (**Figure** **4**A,B). Consistently, stronger fluorescence of the ROS probe was observed in MFC cells in the LI (+) and LLI (+) groups under confocal microscopy (Figure 4C). Furthermore, MFC cells incubated with Icy7 (liposome) showed much higher levels of ROS than those treated with IR780 (liposome) under laser irradiation (Figure S9, Supporting Information). In contrast, without laser irradiation, ROS generation in the cells was similar in all treatment groups (Figure S10, Supporting Information). In response to PDT, CRT translocated from the endoplasmic reticulum to the surface of the plasma membrane, and these DAMPs subsequently induced maturation of DCs and activation of specific T cells to enhance the host antitumor immune response.^[^ ^40^ ^]^ Confocal microscopy showed that both CRT exposure and nuclear efflux of HMGB1 were higher in the LI (+) and LLI (+) groups compared with the PBS (+) and LL (+) groups (Figure 4D; Figure S11, Supporting Information). However, without laser irradiation, no differences were seen in either CRT exposure or nuclear HMGB1 efflux between the different groups (Figure S12, Supporting Information). ATP release and HMGB1 secretion are considered hallmarks of ICD and thus of effective anti‐tumor immune responses.^[^ ^41^ ^]^ Here, ATP and HMGB1 were measured by enzyme‐linked immunosorbent assays (ELISAs). The cells in the LI (+) and LLI (+) groups showed much higher levels of ATP and HMGB1 compared with the PBS (+) and LL (+) groups, and the levels of ATP and HMGB1 were slightly higher in the LLI (+) group compared to the LI (+) group. However, without laser irradiation, both ATP release and HMGB1secretion were similar in the different treatment groups (Figure S13, Supporting Information). As Liproxstatin‐1 was encapsulated into the nanodrugs, the degree of ferroptosis in the cells was determined by flow cytometry. This showed significantly reduced ferroptosis in the LL (+) group compared to other groups (Figure 4E,F; Figure S6H,I, Supporting Information). DAMPs, induced by PDT, may potentially play an important role in stimulating DC maturation. Cells in the LI (+) or LLI (+) groups significantly promoted DC maturation compared with PBS (+) and LL (+) groups, while DC maturation was slightly increased in the LLI (+) group versus the LI (+) group (Figure 4G,H).

### 2.5 Inhibition of Neutrophil Ferroptosis by LLI

**Figure 5:**
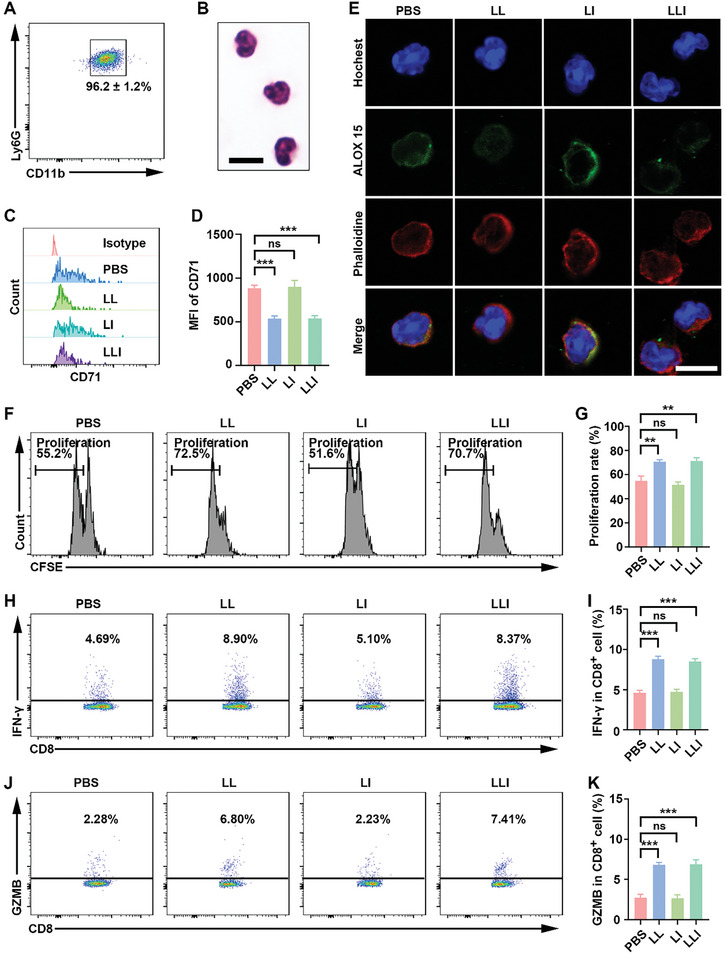
Inhibition of neutrophil ferroptosis enhanced the function of CD8^+^ T cells. A) purity of mouse neutrophils sorted from MFC tumors by flow cytometry, *n* = 3. B) Wright staining of sorted neutrophils. C,D) CD71 expression of neutrophils detected by flow cytometry after various treatments, *n* = 3. E) Fluorescence imaging of ALOX 15 in neutrophils after various treatments (hochest; blue), and (ALOX 15; green) and (phalloidine; red). Scale bar, 25 µm. F,G) Proliferation of CD8^+^ T cells determined by flow cytometry after being cocultured with nanodrug‐pretreated neutrophils, *n* = 3. H,I) IFN‐γ expression of CD8^+^ T cells determined by flow cytometry after being cocultured with nanodrug‐pretreated neutrophils, *n* = 3. J,K) GZMB expression of CD8^+^ T cells determined by flow cytometry after being cocultured with nanodrug‐pretreated neutrophils, n = 3. (Data were presented as the mean ± SD, ^**^ *p* < 0.01, ^***^ *p* < 0.001, and ns, not significant).

It has been reported that ferroptosis‐associated death of neutrophils in the TME leads to the release of oxygenated lipids which reduce the activity of T cells.^[^ ^21^ ^]^ Thus, the degree of ferroptosis of neutrophils within the TME and spleens of tumor‐bearing mice was investigated by flow cytometry. The results showed that intra‐tumoral neutrophils were susceptible to ferroptosis indicated by increased CD71 expression compared with neutrophils in the spleen (Figure S14, Supporting Information). Sorting of the neutrophils in the mouse tumor tissues by flow cytometry with a purity of ≈96% (**Figure** **5**A), and the morphology of isolated neutrophils was observed microscopically after Wright staining (Figure 5B). The isolated neutrophils from mouse tumor tissues incubated with LL and LLI without laser irradiation showed downregulation of CD71, demonstrating inhibition of neutrophil ferroptosis by liproxstatin‐1, and were consistent with the results in HL60 cell line evaluated by Weston Blot (Figure 5C,D; Figure S15, Supporting Information). The presence of ALOX 15 has been found to be critical for ferroptosis,^[^ ^42^ ^]^ and neutrophils incubated with LL and LLI without laser irradiation showed marked downregulation of ALOX15, as shown by CLSM (Figure 5E). To investigate the effects of neutrophil ferroptosis on CD8^+^ T‐cell function, spleen‐derived CD8^+^ T cells were sorted by flow cytometry, resulting in a viability and purity of over 95% (Figure S16, Supporting Information), and were cocultured with neutrophils derived from MFC cell tumors after pre‐incubation with different nanodrugs without laser irradiation. It was found that the proliferation of cytotoxic CD8^+^ T cells was decreased when the neutrophils were incubated with PBS or LI (Figure 5F,G). IFN‐γ and granzyme‐B (GZMB) are effector molecules of CD8^+^ T cells.^[^ ^43^ ^]^ Inhibition of neutrophil ferroptosis significantly increased the expression of both IFN‐γ and GZMB in CD8^+^ T cells (Figure 5H,K).

### 2.6 Tumor Targeting and Biodistribution of LLI in Vivo

**Figure 6:**
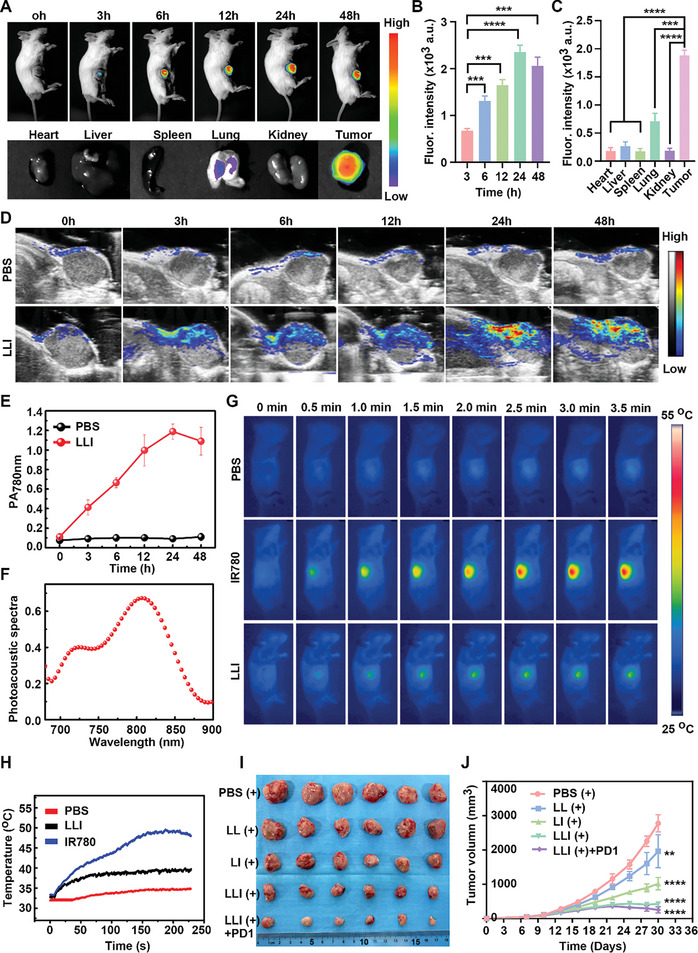
Tumor targeting and synergistic anti‐tumor therapy of LLI. A–C) In vivo fluorescence imaging of MFC tumor‐bearing mice at different time points after LLI injection via tail vein and e*x vivo* fluorescence imaging of main organs and tumors at 48 h after injection. D) Representative photoacoustic (PA) images of tumor regions in living mice at different time points after intravenous injection of LLI (780 nm, 0.5 W cm^−2^). E) Real‐time PA intensity profiles of tumor areas in (D), *n* = 3. F) PA spectra of LLI. G) Thermal images of MFC tumor‐bearing mice at different time points after intravenous injection of different solutions following laser irradiation (808 nm, 0.5 W cm^−2^). H) Tumor temperature changes at different time points after intravenous injection of different solutions following laser irradiation (808 nm, 0.5 W cm^−2^). I) Macroscopic image of tumor tissues from mice receiving various treatments, *n* = 6. J) Tumor growth curve of mice receiving various nanodrugs treatment, *n* = 6. (Data were presented as the mean ± SD, ^**^ *p* < 0.01, ^***^ *p* < 0.001, and ^****^ *p* < 0.0001).

Nano drugs show extravasation and accumulation in tumor tissues due to the presence of inter‐endothelial gaps in the tumor blood vessels.^[^ ^44^ ^]^ Fluorescence imaging was performed to investigate the retention and biodistribution of LLI in MFC tumor‐bearing mice. The in vivo fluorescence imaging showed that the fluorescence intensity in the tumors increased gradually, reaching a maximum at 24 h after intravenous injection via the tail vein (**Figure** **6**A,B), indicating the optimal time point for performing laser irradiation. Forty‐eight hours after administration, the fluorescence signal of LLI remained strong, indicating the effective accumulation of LLI in the MFC tumor in vivo. Furthermore, at 48 h after LLI administration, the *ex vivo* fluorescence intensity of the tumors was much higher than that of the heart, liver, spleen, lung, and kidney, demonstrating the favorable accumulation of LLI at the tumor site for the targeted regulation of the tumor immune microenvironment (TIME) (Figure 6C). As a near‐infrared fluorescence imaging dye, photoacoustic (PA) imaging was employed to monitor the dynamic accumulation of LLI at the tumor site in real‐time.^[^ ^45^ ^]^ The PA_780 nm_ signal of LLI in the tumor region increased gradually, reaching a maximum at 24 h after intravenous administration (Figure 6D–F). Moreover, heptamethine cyanine dyes can serve as typical photothermal agents to promote increased temperature following laser irradiation;^[^ ^46^ ^]^ thus, photothermal imaging was conducted to evaluate the effects of IR780 and Icy7 in vivo. After treatment of mice with IR780, markedly increased tumor temperatures were observed, reaching a maximal tumor temperature of 49.3 °C. The temperature of Icy7‐treated mice increased slightly, with a final tumor temperature of 39.1 °C (Figure 6G,H).

### 2.7 Antitumor Effects in Vivo

Under laser irradiation, the photosensitizer generated ROS to induce ICD in tumor cells, enhancing antigen presentation by APCs.^[^ ^15^ ^]^ In addition, neutrophil ferroptosis has been found to cause immune suppression in tumors, and pharmacological inhibition of ferroptosis led to tumor regression and exhibited a synergistic therapeutic effect with anti‐PD‐1 antibodies.^[^ ^21^ ^]^ The synergistic antitumor potential of codelivered Icy7 and Liproxstatin‐1 was investigated (Figure S17A, Supporting Information). The tumors were harvested from mice at the end of the study and were imaged (Figure 6I). A higher degree of tumor growth was observed in mice treated with PBS (+), with the tumor volumes in the control group reaching ≈3000 mm^3^ at 20 days after treatment. The different nanodrugs all exhibited inhibitory effects on tumor growth. In the combined LL (+) and LI (+) treatments, LLI (+) administration resulted in a marked reduction in tumor growth. Treatment with LLI (+) combined with anti‐PD‐1 antibody was the most effective in reducing tumor growth, which clearly demonstrated that Icy7 and Liproxstatin‐1 acted synergistically and thus contributed to the efficacy of anti‐PD‐1 therapy (Figure 6J). Compared to the LLI (+) group, treatment with LLI without laser irradiation resulted in similar effects to LL (+), which indicated that the antitumor action of the photosensitizer (Icy7) requires laser irradiation (Figure S18A–C, Supporting Information). Treatment with anti‐PD‐1 antibody alone only slightly inhibited tumor growth of MFC cells, without statistical significance (Figure S18D‐F, Supporting Information). Consistently, the tumor weights in the group treated with both LLI (+) and the anti‐PD‐1 antibody were lower than those in the other groups (Figure S17B, Supporting Information). To evaluate the effects of nanodrugs on tumor cell proliferation and apoptosis, sections of tumor tissues from each group were stained with H&E, Ki67, and TUNEL. This showed that the combination of LLI (+) with the anti‐PD‐1 antibody resulted in the highest rate of apoptosis and the lowest level of proliferation (Figure S17C–E, Supporting Information), indicating that PDT acted synergistically with ferroptosis inhibition to contribute to anti‐PD‐1 therapy.

### 2.8 Biosafety Evaluation

To evaluate the side effects of nanodrugs in vivo, the body weights of mice were recorded every other day, and the blood and main organs of the mice were collected for further analysis at the end of the experiments. None of the groups showed any significant loss in body weight (Figure S17F, Supporting Information), and no obvious pathological changes were observed in the major organs of the treated mice, including the heart, liver, spleen, lung, and kidney (Figure S19A, Supporting Information). Blood biochemical analyses, including indicators of liver function (ALT and AST) and renal function (Cr and BUN), were at normal levels and showed no significant differences between the groups (Figure S19B–D, Supporting Information). These results indicated that LLI had low side effects in vivo.

### 2.9 Nanodrug‐Mediated TIME Remodeling

**Figure 7:**
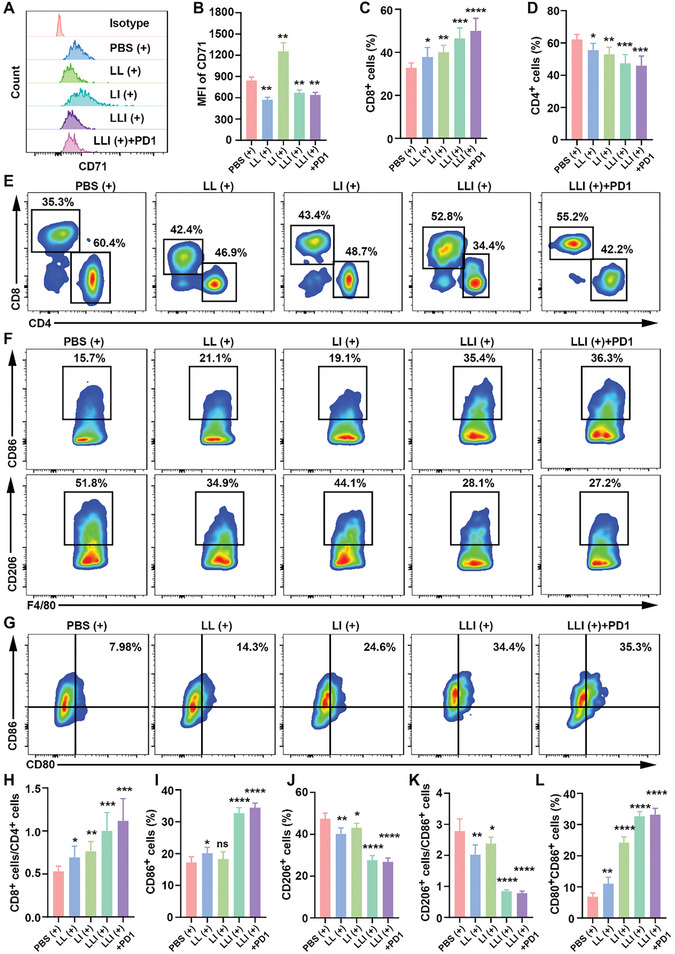
Nano drugs enhanced anti‐tumor immune responses in MFC tumors. A,B) CD71 expression of neutrophils in tumor tissues determined by flow cytometry after different treatments (gated on CD11b^+^Ly6G^+^ cells), *n* = 6. C–E) The percentage of CD4^+^ and CD8^+^ T cells in tumor tissues was determined by flow cytometry after different treatments (gated on CD3^+^ T cells), *n* = 6. F) The percentage of M1 and M2 TAMs in tumor tissues was determined by flow cytometry after different treatments (gated on F4/80^+^ cells), *n* = 6. G) The percentage of mature DCs in tumor tissues determined by flow cytometry after different treatments (gated on CD11c^+^ cells), *n* = 6. H) Statistical analysis of the ratios of CD8^+^ to CD4^+^ T cells in different groups, *n* = 6. I–K) Statistical analysis of the percentage of M1 TAMs, M2 TAMs, and the ratios of M2 to M1 TAMs in different groups, *n* = 6. L) Statistical analysis of DC maturation (CD80^+^CD86^+^ cells) in different groups, *n* = 6. (Data were presented as the mean ± SD, ^*^ *p* < 0.05, ^**^ *p* < 0.01, ^***^ *p* < 0.001, and ^****^ *p* < 0.0001).

To better understand the underlying reasons for the effectiveness of the LLI (+) treatment, immune cell infiltration of tumor tissues and spleens was investigated, assessing the levels of neutrophils, T‐cell subpopulations, TAMs, DCs, and effector memory T cells. Gating strategies were used to investigate various immune cells in the tumors and spleens of mice (Figures S20 and S21, Supporting Information). Ferroptosis of neutrophils is reported to induce tumor immune suppression, while inhibition of ferroptosis counteracts the immunosuppressive effects of tumor neutrophils within TME.^[^ ^21^ ^]^ Therefore, neutrophil ferroptosis was evaluated, showing its significant inhibition after treatment with LL (+), LLI (+), and LLI (+) combined with an anti‐PD‐1 antibody (**Figure** **7**A,B). In contrast, neutrophil ferroptosis was higher in the LI (+) group compared with the PBS (+) group, which may be related to the greater generation of ROS within the TME of the LI (+) group. Additionally, ICD induced by PDT could help turn “cold” tumors into “hot” tumors to promote the intra‐tumoral infiltration of cytotoxic T lymphocytes (CTLs).^[^ ^47^ ^]^ The ratios of CD8^+^ to CD3^+^ T cells were found to be significantly increased in the LLI (+) and LLI (+) combined with anti‐PD‐1 antibody groups compared with the PBS (+), LL (+), and LI (+) groups, and the LLI (+) combined with anti‐PD‐1 antibody treatment further increased the infiltration of CD8^+^ T cells (Figure 7C,E). CD4^+^ and CD8^+^ T cells are the main subpopulations of CD3^+^ T cells, and the proportion of CD4^+^ T cells in relation to CD3^+^ T cells was markedly decreased in the groups treated with LLI (+) and LLI (+) together with anti‐PD‐1 antibody (Figure 7D). In addition, the ratio of CD8^+^ to CD4^+^ T cells was significantly elevated in the LLI (+) and LLI (+) combined with anti‐PD‐1 antibody groups (Figure 7H). The recruitment and activation of macrophages by PDT potentiates immunity.^[^ ^48^ ^]^ LLI (+) and LLI (+) combined with anti‐PD‐1 antibody groups resulted in significant decreases in M2 TAMs and increases in M1 TAMs (Figure 7F,I,J). Furthermore, the ratio of CD206^+^ to CD86^+^ cells decreased significantly after these treatments (Figure 7K). DAMPs induced by PDT promoted DC maturation, allowing antigen presentation by the mature DCs and thus activating adaptive immunity.^[^ ^49^ ^]^ Thus, treatment with LLI (+) and LLI (+) combined with anti‐PD‐1 antibody significantly increased the expression of co‐stimulatory molecules CD80 and CD86 in DCs (Figure 7G,L).

### 2.10 Synergistic Immunotherapy of LLI

**Figure 8:**
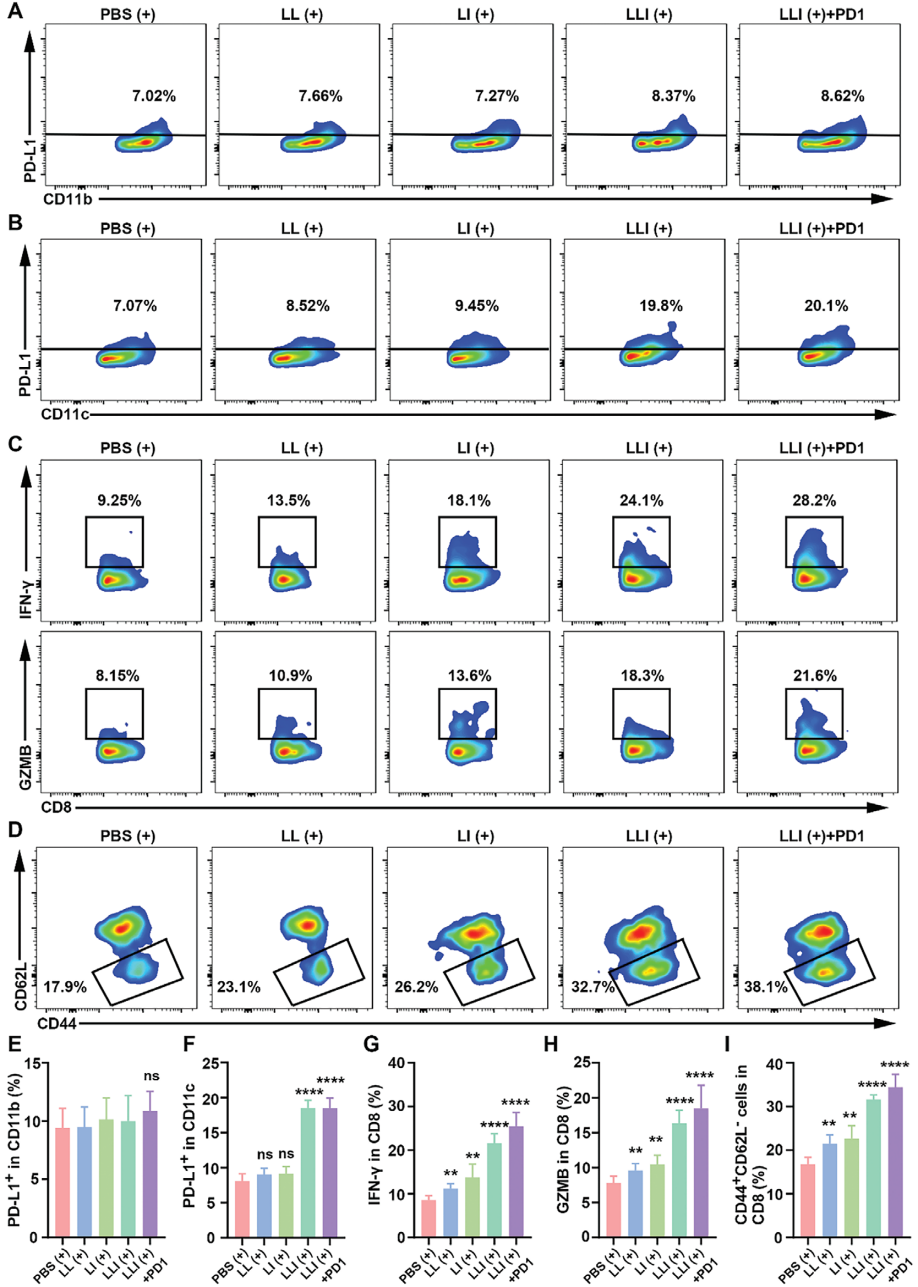
The synergistic effect of nanodrug contributed to the anti‐PD‐1 antibody treatment. A) PD‐L1 expression on CD11b^+^ cells in tumor tissues determined by flow cytometry after different treatments (gated on CD11b^+^ cell), *n* = 6. B) PD‐L1 expression on CD11c^+^ cells in tumor tissues determined by flow cytometry after different treatments (gated on CD11c^+^ cell), *n* = 6. C) Production of IFN‐γ and GZMB in CD8^+^ T cells determined by flow cytometry after different treatments (gated on CD8^+^ T cell), *n* = 6. D) The percentage of effector memory T (TEMs) cells in spleens determined by flow cytometry after different treatments (gated on CD8^+^ T cell), *n* = 6. E,F) Statistical analysis of the percentage of PD‐L1 on CD11b^+^ cells and CD11c^+^ cells in tumor tissues after different treatments, *n* = 6. G,H) Statistical analysis of the percentage of IFN‐γ and GZMB in CD8^+^ T cells after different treatments, *n* = 6. I) Statistical analysis of the percentage of TEMs in spleens after different treatments, *n* = 6. (Data were presented as the mean ± SD, ^**^ *p* < 0.01, ^****^ *p* < 0.0001, and ns, not significant).

Inhibition of ferroptosis has been shown to contribute to tumor regression and exhibit a synergistic therapeutic effect with anti‐PD‐1 antibodies,^[^ ^21^ ^]^ while the combination of PDT and PD‐1 antibody treatment is effective for controlling tumor growth.^[^ ^50^ ^]^ A key player in immunotherapy appears to be the interaction between PD‐1 antigen and its ligand PD‐L1, and the expression of PD‐L1 in immune cells has been found to correlate with the response to anti‐PD‐1 antibody treatment.^[^ ^51^ ^]^ LLI (+) treatment resulted in a significant upregulation of PD‐L1 expression in DCs, together with a slight increase of PD‐L1 expression in CD45^+^CD11b^+^ cells (**Figure** **8**A,B,E,F). Blocking the PD‐1 interaction augments the effector phase of CD8^+^ T cells and increases the production of effector molecules.^[^ ^52^ ^]^ The combination of LLI (+) and anti‐PD‐1 antibody significantly increased the expression of both IFN‐γ and GZMB (Figure 8C,G,H). The generation of immune memory is critical for long‐lasting anti‐tumor immune responses and the prevention of tumor recurrence.^[^ ^53^, ^54^ ^]^ LLI (+) and LLI (+) combined with anti‐PD‐1 antibody treatments showed much higher percentages of TEMs (CD3^+^CD8^+^CD44^+^CD62L^−^), and LLI (+) combined with anti‐PD‐1 antibody treatment further increased TEM levels compared with the LLI (+) treatment (Figure 8D,I).

### 2.11 Nanodrug Treatments with Abscopal Effects

PDT has been reported to have abscopal effects with distant tumors resulting from the induction of systemic antitumor immune responses.^[^ ^33^, ^55^ ^]^ Therefore, we investigated the growth of untreated distant tumors following treatment of primary tumors by different nanodrugs with laser irradiation. Treatment with LLI (+) and LLI (+) combined with anti‐PD‐1 antibody showed a marked inhibition of distant tumor growth (Figure S22A‐F, Supporting Information). To evaluate the effects of nanodrugs on distant tumor proliferation and apoptosis, sections of distant tumors were stained with H&E, Ki67, and TUNEL. The combination of LLI (+) with anti‐PD‐1 antibody led to the highest rate of apoptosis together with the lowest proliferation rate (Figure S22G‐I, Supporting Information). Furthermore, the TIMEs of distant tumors were also evaluated by FACS. Neutrophil ferroptosis in the distant tumors was significantly reduced after treatment with LL (+), LLI (+), and LLI (+) combined with anti‐PD‐1 antibody. whereas, neutrophil ferroptosis in distant tumors did not differ significantly between the PBS (+) and LI (+) groups (Figure S23A,B, Supporting Information), which was correlated with the absence of light irradiation in distant tumors. The ratios of CD8^+^ to CD3^+^ T cells in distant tumors were significantly increased in the groups treated with LLI (+) and LLI (+) combined with anti‐PD‐1 antibody compared with the PBS (+), LL (+), and LI (+) groups, and treatment with LLI (+) combined with anti‐PD‐1 antibody further increased the infiltration of CD8^+^ T cells (Figure S23C,E, Supporting Information). The proportion of CD4^+^ cells among CD3^+^ T cells within distant tumors showed an obvious decrease in the group treated with LLI (+) combined with anti‐PD‐1 antibody (Figure S23D, Supporting Information). In addition, the ratios of CD8^+^ to CD4^+^ T cells in distant tumors were significantly elevated in LLI (+) and LLI (+) combined with anti‐PD‐1 antibody groups (Figure S23H, Supporting Information). Furthermore, treatment with LLI (+) and LLI (+) together with anti‐PD‐1 antibody resulted in significant decreases in M2 TAMs and increases in M1 TAMs in the distant tumors (Figure S23F,I,J, Supporting Information). In addition, the ratios of CD206^+^ to CD86^+^ cells in distant tumors were significantly reduced after treatment with LLI (+) and LLI (+) combined with anti‐PD‐1 antibody (Figure S23K, Supporting Information). Simultaneously, LLI (+) and LLI (+) combined with anti‐PD‐1 antibody treatments significantly increased the expression of the co‐stimulatory molecules CD80 and CD86 by DCs in distant tumors (Figure S23G,L, Supporting Information). Importantly, treatment with LLI (+) together with anti‐PD‐1 antibody induced higher expression of both IFN‐γ and GZMB in the distant tumors (Figure S24, Supporting Information). These results demonstrated that the combined treatment of LLI (+) and anti‐PD‐1 antibody induced significantly stronger systemic antitumor immunity not only against primary tumors but also inhibit distant tumors.

## 3 Discussion

In this study, we investigated ferroptosis in different cell types within TME in the context of the pathophysiology of GC, finding that neutrophils were susceptible to ferroptosis resulting in the creation of an immunosuppressive TME in GC. Although ferroptosis reduced the neutrophil numbers within the TME, the release of immunosuppressive oxidized lipids by ferroptosis cells had inhibitory effects on T cells.^[^ ^21^ ^]^ This suggests that neutrophil ferroptosis could be a potential therapeutic target for GC. PDT, as an emerging therapeutic modality, has been extensively investigated and shown to play a vital role in current cancer therapy. The production of ROS is vital for effectual PDT in cancer treatment. Di‐iodinated cyanine has a significantly higher efficiency for ^1^O_2_ production,^[^ ^18^ ^]^ and the two iodine atoms were successfully incorporated into IR780 (Icy7) for enhanced PDT. Icy7 was found to generate significant ROS production compared to IR780 under 808 nm laser irradiation. In addition, neutrophil ferroptosis can be triggered by increased ROS generation within TME. Here, a nano drug encapsulating both the ferroptosis inhibitor Liproxstatin‐1 and the modified photosensitizer Icy7 was constructed to induce ICD in tumor cells, remodel immunosuppressive TME, and work in synergy with anti‐PD‐1 antibody treatment.

In vitro, LLI was shown to be taken up by MFC cells to generate ROS under laser irradiation, with the induced ROS promoting apoptosis of tumor cells and triggering ICD resulting in the release of tumor‐associated antigens and subsequent activation of immune cells.^[^ ^56^ ^]^ The levels of ATP and HMGB1 were observed to be slightly higher in cells of LLI (+) group compared to the LI (+) group, which indicated that LLI (+) effectively elicited ICD in tumor cells. It is possible that the increased levels of cell death following LLI (+) treatment may be associated with increases in other forms of cell death rather than ferroptosis. It was found that LLI (+) treatment of MFC cells resulted in more effective promotion of DC maturation than other nano‐drugs. Neutrophils within TME of GC tumors spontaneously died by ferroptosis, and neutrophils in the spleens and tumors of MFC tumor‐bearing mice were then investigated. Consistent with the results of clinical studies on GC, neutrophil ferroptosis in MFC tumors was significantly increased relative to that of neutrophils in the spleens. Neutrophils from the MFC tumors were then sorted by flow cytometry and incubated with the different nano‐drugs. Neutrophil ferroptosis was found to be inhibited in the LL and LLI groups compared to the LI and PBS groups, which indicated that the ferroptosis of neutrophils was inhibited by Liproxstatin‐1. Oxidized lipids from ferroptosis cells limit the functions of the T cells,^[^ ^21^ ^]^ thus, CD8^+^ T cells were cocultured with tumor neutrophils after pretreatment with different nano‐drugs. The results showed that inhibition of neutrophil ferroptosis alleviated the suppression of CD8^+^ T cell function by oxidized lipid.

In vivo, fluorescence imaging was performed to investigate the retention and biodistribution of LLI in MFC tumor‐bearing mice. The results showed a specific accumulation of LLI in the tumor sites. This targeting phenomenon is commonly referred to as the enhanced permeability and retention (EPR),^[^ ^57^ ^]^ and provides a basis for the targeted regulation of TME. Additionally, LLI was also shown to accumulate specifically in tumors, as measured by photoacoustic imaging (PAI). This indicated that Icy7‐loaded nanoparticles not only allowed PA imaging but also NIR fluorescence imaging, thus providing more anatomical information about tumors. Compared with IR780, the temperature of Icy7 in the tumor site was significantly reduced with the temperature decreasing from ≈50 to 40 °C. However, moderate temperature increases of a few degrees above body temperature (≈40–43 °C) stimulated the immune response, thus enhancing anti‐tumor treatment.^[^ ^58^ ^]^ In terms of the combination of LL (+) and LI (+) treatments, LLI (+) treatment showed an obvious inhibitory effect on tumor growth. On the one hand, PDT effectively induced photodynamic immunogenic cancer cell death. On the other hand, inhibition of neutrophil ferroptosis remodeled the TME to reduce suppression of CD8^+^ T cell function. Given the obvious inhibitory effect on tumor growth, LLI may exert synergistic effects by the combination of PDT with ferroptosis inhibition. Hence, LLI has potential for application in the diagnosis and treatment of tumors.

The development of tumor immunotherapy has brought hope for the cure of tumors. The most common ICBs refer to blocking immune inhibitory receptors CTLA4 and PD‐1 on T cells, or PD‐L1 on tumor cells and tumor‐infiltrating immune cells using antagonistic antibodies. However, ≈20% of patients, including those with GC, are unresponsive to currently used ICBs.^[^ ^59^ ^]^ This is partly due to a lack of antitumor immune cells and immune checkpoints, as well as an immunosuppressive TME. Considering the ability of PDT‐mediated ICD to promote the release of DAMPs, it is tempting to speculate that PDT could prime cancer immunotherapy to increase the response rate. However, the efficacy of PDT is severely limited by tumor hypoxia.^[^ ^60^ ^]^ As IR780 targets the mitochondria, it can alleviate hypoxia by influencing mitochondrial respiration.^[^ ^61^ ^]^ Besides, moderate temperatures also promote both blood circulation and oxygen supply at the tumor site.^[^ ^62^ ^]^ Thus, LLI can both relieve the hypoxic environment of the tumor and stimulate a greater generation of ROS. Moreover, inhibition of neutrophil ferroptosis increased the proportions of effector CD8^+^ T cells, natural killer cells, and resident memory cells in TME, and synergy between Liproxstatin‐1 treatment and blocking PD‐1 was also observed.^[^ ^21^ ^]^ LLI (+) combined with anti‐PD‐1 antibody treatment was found to be most effective in inhibiting tumor growth, which clearly demonstrated that Icy7 and Liproxstatin‐1 acted synergistically to contribute to anti‐PD‐1 antibody therapy. Meanwhile, the upregulation of PD‐L1 is regarded as an important response in predicting the effectiveness of immunotherapy.^[^ ^63^ ^]^ LLI (+) treatment significantly increased the expression of PD‐L1 on DCs, thus priming DC activation and the response to immunotherapy. IFN‐γ and GZMB released by CD8^+^ T cells are also critical drivers in immunotherapy.^[^ ^64^ ^]^ The combination of LLI (+) and anti‐PD‐1 antibody increased the release of IFN‐γ and GZMB compared to the other groups. The growth of distant tumors in LLI (+) combined with anti‐PD‐1 antibody group was also markedly inhibited. This may be associated with LLI‐based immunogenic PDT and neutrophil‐targeting immunotherapy to synergistically boost anti‐PD‐1 antibody treatment to elicit potent systemic antitumor immunity with abscopal effects.

In conclusion, neutrophils were found to be susceptible to ferroptosis, thus creating an immunosuppressive TME in GC, and suggesting that this may represent a potential therapeutic target for GC. Heavy atoms were incorporated into the photosensitizer IR780 (Icy7) leading to greater ROS production to enhance the effect of PDT. A nanodrug incorporating both ferroptosis inhibitor Liproxstatin‐1 and modified photosensitizer Icy7 was constructed for use as a synergistic PDT and neutrophil‐targeting immunotherapy for GC. This nanodrug was shown to have good bio‐compatibility and excellent tumor‐targeting properties. LLI was shown to be internalized by MFC cells to generate ROS under laser irradiation at 808 nm, triggering ICD. During this process, immunostimulatory signals, including CRT, played a crucial role in promoting the recruitment of APCs and T‐cell infiltration. Simultaneously, liposome‐delivered Liproxstatin‐1 effectively inhibited ferroptosis of neutrophils. This led to a reduction in the levels of oxidized lipids, consequently restoring the function of T cells. This synergistic effect of LLI‐mediated immunogenic PDT combined with neutrophil‐targeting immunotherapy significantly inhibited tumor growth. This synergistic approach was achieved by promoting the maturation of DCs, increasing infiltration of CD8^+^ T cells in tumors, and inducing expression of TEMs in the spleen. Furthermore, this treatment also effectively decreased infiltration of M2‐like TAMs. Moreover, the combined synergistic treatment was found to increase the expression of PD‐L1 on DCs, contributing to the treatment response of ICBs and further suppressing tumor growth when combined with anti‐PD‐1 antibody. Notably, LLI reduced TME immunosuppression, and elicited potent TME and systemic antitumor immunity with immunological memory. This approach resulted in the regression of both local and distant tumors, achieving an abscopal effect. These results highlighted the significant potential of LLI for the treatment of GC.

## 4 Experimental Section

### Gastric Cancer Samples

A total of 10 human GC samples, including paired tumor tissues, normal adjacent tissues, and peripheral blood, were obtained from patients who underwent surgery in the Affiliated Drum Tower Hospital of Nanjing University Medical School. All specimens were obtained after written informed consent from patients (reference number: 2021‐361‐02). Patients with preoperative anti‐tumor treatments (neoadjuvant radiotherapy or chemotherapy) were excluded. Polymorphprep (Axis‐Shield, Norway) was utilized for the isolation of polymorphonuclear leukocytes from human peripheral blood. Freshly resected human GC tissues were minced by scissors into 2–4 mm diameter pieces and digested in 2.5 mg mL^−1^ collagenase IV and DNase. Percoll (Cytiva, USA) was used to isolate tumor‐infiltrating neutrophils.

### Flow Cytometry

The cells isolated from human GC samples were then stained with fluorochrome‐conjugated anti‐human antibodies against CD45‐PE, CD11b‐FITC, CD15‐PE/Cy7, CD71‐APC, CD3‐FITC, CD4‐PerCP/Cy5.5, CD8‐BV605, CD68‐PE/Cy7, CD86‐PerCP/Cy5.5, CD206‐BV421, CD11c‐BV421, and CD80‐FITC for 30 min at 4 °C. Cells were then washed three times with PBS and analyzed by FACS Aria Cytometer (BD Biosciences, Franklin Lakes, NJ, USA). Data were collected as FCS files. The antibodies for flow cytometric analysis in this part were purchased from BioLegend (San Diego, CA, USA). All reagents were used according to the manufacturer's specifications.

### Immunofluorescence Staining

Neutrophils were isolated from human peripheral blood and GC tissues by flow cytometry and then centrifuged onto the slide in 24‐well plates. After fixation with 4% paraformaldehyde and permeabilization by Triton‐100, nonspecific binding was blocked using 10% bovine serum albumin (BSA) for 30 min. The cells were then stained with anti‐CD71 (Abcam, USA) antibody overnight at 4 °C. Following incubation with a secondary antibody conjugated with Alexa Fluor 488, cells were observed by a confocal laser scanning microscope (CLSM, OLYMPUS, Japan).

### Reagents, Synthesis, and Characterization of Nanoparticles

DSPE‐PEG2000 and cholesterol (CHO) were purchased from Shanghai Yuanye Bio‐Technology (Shanghai, China). Lecithin (PC) and Liproxstatin‐1 were purchased from MedChemExpress (Shanghai, China). As ROS production can be increased by the incorporation of heavy atoms into the photosensitizer, di‐iodinated IR780 (Icy7) was synthesized for enhanced PDT (Supporting Information). All reagents used in the study were analytical grade. For the synthesis of nanodrugs co‐loaded with Liproxstatin‐1 and Icy7 (LLI), a total of 40 mg of PC:Chol:DSPE‐PEG2000 (with a molar ratio of 50:25:5) were utilized to prepare liposome. 1 mg Icy7 and 36 µg Liproxstatin‐1 were sufficiently dissolved in 10 mL of dichloromethane in the dark environment. A rotary evaporation procedure (42 °C, 100 rpm, 20 min) was performed to remove solvent and form lipid films. 5 ml of ultrapure water was added to peel off the lipid films. Suitable particle sizes of the nanoparticles were obtained using a probe supersonic method (power level 130 W, 20 kHz, 10 min). The nanodrug solution was purified by using a 30 KDa Amicon Ultra filter under centrifugation at 2000 g for 5 min to remove free Icy7 and Liproxstatin‐1. To explore the release of Icy7 and Liproxstatin‐1 from nanodrugs, both DiO (donor, Ex/Em 488/501 nm) and DiI (acceptor, Ex/Em 501/565 nm) were incorporated into the LLI. When nanodrugs were excited at 488 nm, it was expected that they would give a red fluorescence from DiI because of the energy transfer from the donor to the acceptor within the Forster distance (Forster resonance energy transfer, FRET).^[^ ^65^ ^]^ When the DiO and DiI were released from liposomes, red fluorescence decreased duo to the longer donor‐acceptor distance. Nano drugs loaded with single Liproxstatin‐1 (LL) or Icy7 (LI) were then prepared accordingly. The encapsulation efficiency of Icy7 and Liproxstatin‐1 was determined by UV‐vis spectrometry and high‐performance liquid chromatography (HPLC), respectively. Furthermore, inductively coupled plasma‐mass spectrometry (ICP‐MS) was also performed to quantify the concentration of Icy7 in nanodrugs.^[^ ^66^ ^]^ The morphology and size of NPs were evaluated by transmission electron microscope (TEM, JEOL, Japan). The mean particle diameter, size distribution, polydispersity index (PDI), and Zeta potential were determined by dynamic light scattering (DLS, Nanobrook Omni, Brookhaven, USA). UV‐vis spectrometry was used to measure the absorption spectra of the free Icy7 and nanodrugs, and fluorescence spectra of the free Icy7 and nanodrugs were measured using a Horiba FluoroMax‐4 spectrofluorometer at 808 nm excitation. The photosensitivity of IR780 and Icy7 for ^1^O_2_ generation was measured by determining the absorbance spectra of 1,3‐diphenylisobenzofuran (DPBF), with the decrease in absorbance of DPBF corresponding to the production of ^1^O_2_ under 808 nm light irradiation. An infrared thermal imaging system was used for recording the temperature changes of IR780 and Icy7 solutions, and real‐time temperatures of solutions were recorded using an FLIR thermal mapping camera under 808 nm light irradiation (0.5 W cm^−2^). To explore the stability of nanodrugs, 0.1 mL of the nanoparticle solution was added to 1.9 mL PBS containing 10% fetal bovine serum (FBS) (pH = 7.4, 37 °C), and the diameters of the nanoparticles were measured at different time points using DLS.

### Dark and Light Toxicities

MFC gastric cancer cells were cultured in Roswell Park Memorial Institute (PRMI) 1640 medium with 10% FBS and 1% penicillin/streptomycin at 37 °C in a 5% CO_2_ atmosphere. The cytotoxicity of nanodrugs with or without light irradiation was determined via CCK‐8 assay. Briefly, MFC cells were seeded into 96‐well plates at a density of 8 × 10^3^ cells/well and incubated overnight. Cells were then treated with various concentrations of LL, LI, and LLI for 6 h. After washes with PBS, cells were or were not exposed to laser irradiation at 808 nm for 1 min (0.5 W cm^−2^) and cultured for another 24 h. Cell viability was evaluated by a CCK‐8 kit (Beyotime Biotechnology, China) according to the manufacturer's protocol.

### Apoptosis of Tumor Cells

To analyze cell apoptosis, MFC cells were seeded into 24‐well plates at a density of 1 × 10^5^ cells/well and incubated overnight. Cells were then treated with PBS, LL, LI, and LLI at the identical concentration of Icy7 (2.5 µM) for 6 h. After washes with PBS, cells were or were not exposed to laser irradiation at 808 nm for 1 min (0.5 W cm^−2^) and cultured for another 24 h. Besides, 3D tumor spheroid models were widely used in mimicking TME in vivo,^[^ ^38^ ^]^ and MFC cells were seeded into 96‐well clear round bottom ultra‐low attachment plates at the cell number of 2000/well. Annexin V‐FITC/propidium iodide (KeyGEN Biotech, China) and JC‐1 assay kit (Beyotime, China) were used for detecting tumor cell apoptosis and mitochondria function, respectively.

### Live/Dead Viability Assay

MFC cells were seeded into 24‐well plates at a density of 1 × 10^5^ cells/well and incubated overnight. Cells were then treated with PBS, LL, LI, and LLI at the identical concentration of Icy7 (2.5 µM) for 6 h. After washes with PBS, cells were or were not exposed to laser irradiation at 808 nm for 1 min (0.5 W cm^−2^) and cultured for another 24 h. Cells were harvested and stained with Calcein AM (2 µM) and PI (2 µM) (KeyGEN Biotech, China), and were detected by confocal microscopy.

### Detection of ROS Generation

MFC cells were seeded into 24‐well plates at a density of 1 × 10^5^ cells/well and incubated overnight. Following treated with PBS, LL, LI, and LLI at the identical concentration of Icy7 (2.5 µM) for 6 h, cells were incubated with DCFH‐DA (Beyotime Biotechnology, China) for 30 min at 37 °C. Then, cells were or were not exposed to laser irradiation for 1 min (0.5 W cm^−2^), and ROS generation was determined by flow cytometry and confocal microscopy.

### Determination of ICD Biomarker

MFC cells with a density of 1 × 10^4^ were seeded into 35 mm dish and incubated overnight. The cells were then treated with PBS, LL, LI, and LLI at the identical concentration of Icy7 (2.5 µM) for 6 h. After washes with PBS, cells were or were not exposed to laser irradiation at 808 nm for 1 min (0.5 W cm^−2^) and cultured for another 24 h. After fixation with 4% paraformaldehyde and permeabilization by Triton‐100, nonspecific binding was blocked using 10% BSA. The cells were stained with antibodies of anti‐mouse CRT (Abcam, USA) and HMGB1 (Abcam) overnight at 4 °C. Following incubation with a secondary antibody conjugated with Alexa Fluor 488 or 594, cells were observed by CLSM. To detect the release of HMGB1 and ATP, MFC cells were seeded into 24‐well plates at a density of 1 × 10^5^ cells/well and incubated overnight. The cells were then treated with PBS, LL, LI, and LLI at the identical concentration of Icy7 (2.5 µM) for 6 h. After washes with PBS, cells were or were not exposed to laser irradiation at 808 nm for 1 min (0.5 W cm^−2^) and cultured for another 24 h. Cell supernatants were collected and determined using HMGB1 and ATP ELISA Kit (MEIMIAN, China) according to the manufacturer's instructions.

### DC Maturation

MFC cells were seeded in 24‐well plates at a density of 1 × 10^5^ cells/well and were incubated overnight. The cells were then treated with PBS, LL, LI, and LLI at the identical concentration of Icy7 (2.5 µM) for 6 h. After washing with PBS, cells were exposed to 808 nm laser irradiation for 1 min (0.5 W cm^−2^). Eight‐week‐old BALB/c mice were sacrificed, and the bone marrow was flushed out into cold PBS and then passed through a 70‐µm cell strainer to remove cell clumps and other tissues. Following incubation with RBC lysis solution to remove red blood cells, mouse bone marrow‐derived DCs (BMDCs) were cocultured with nanodrug‐pretreated MFC cells for 48 h. The levels of the co‐stimulatory molecules CD80 and CD86 in BMDCs were evaluated using flow cytometry.

### Ferroptosis of Neutrophils in Mouse

Five‐week‐old male BALB/c mice were purchased from Gempharmatech Co., Ltd (Nanjing, China) and inoculated with MFC cells to obtain tumor‐bearing mice according to the protocols of an animal model. Three weeks after MFC cell inoculation, mice were sacrificed, and spleens and tumors were collected, and processed into single‐cell suspensions. The cells from both spleens and tumors were stained with fluorochrome‐conjugated antibodies of anti‐mouse CD45‐APC/Cy7, CD11b‐FITC, Ly6G‐PE, and CD71‐PE/Cy7 for 30 min at 4 °C. The cells were then washed three times in PBS and analyzed by flow cytometry.

### Neutrophil and CD8^+^ T Cell Purification

Single‐cell suspensions of spleens and tumor tissues from MFC tumor‐bearing mice were stained with fluorochrome‐conjugated antibodies against mouse CD45‐APC/Cy7, CD11b‐FITC, Ly6G‐PE, CD71‐PE/Cy7, CD3‐PE/Cy7, CD4‐APC, and CD8‐BV510 for 30 min at 4 °C. CD8^+^ T cells (gated as CD3^+^CD8^+^ cells) from spleens and neutrophils (gated as CD11b^+^Ly6G^+^ cells) from tumors were sorted with BD FACS Aria and counted.

### Inhibition of Neutrophil Ferroptosis in Vitro

Neutrophils were isolated from tumor tissues of MFC tumor‐bearing mice by flow cytometry and were seeded into 48‐well plates at a density of 1 × 10^5^ cells/well and incubated overnight. The cells were then treated with PBS, LL, LI, and LLI with the same concentration of Liproxstatin‐1 (0.5 µM) without irradiation for 24 h. Cells were washed with PBS and stained with fluorochrome‐conjugated anti‐mouse antibodies against CD45‐APC/Cy7, CD11b‐FITC, Ly6G‐PE, and CD71‐PE/Cy7 for 30 min at 4 °C. Ferroptosis of neutrophils was evaluated by flow cytometry. Meanwhile, neutrophils isolated from tumor tissues were seeded into 24‐well plates (1 × 10^5^ cells/well) with slides and incubated overnight. The cells were treated with PBS, LL, LI, and LLI at the same concentration of Liproxstatin‐1 (0.5 µM) without irradiation for 24 h. After fixation with 4% paraformaldehyde and permeabilization with Triton‐100, nonspecific binding was blocked using 10% BSA. The cells were then incubated overnight at 4 °C with the primary antibodies against CD71 (Abcam, USA) and ALOX 15 (Abcam). Following incubation with a secondary antibody conjugated with Alexa Fluor 488, cells were observed by CLSM. The promyelocytic leukemia cell line (HL60) was used to verify the relative protein expression, and HL60 cells were seeded into 24‐well plates (1 × 10^5^ cells/well) and incubated overnight. Nanodrugs were cocultured with Erastin‐pretreated (20 µM, 12 h) HL60 cells for 24 h.^[^ ^67^ ^]^ After treatments with nanodrugs without laser irradiation, total protein was extracted, and the protein concentration was detected by a BCA kit. After separation on 10% SDS‐PAGE gel, proteins were transferred to polyvinylidene difluoride (PVDF) membranes and incubated with an anti‐CD71 antibody (Abcam, USA), anti‐ACSL4 (Abcam), and anti‐GPX4 (Abcam). The proteins were then visualized using an ECL kit.

### CD8^+^ T Cell Function

CD8^+^ T cells isolated from spleens were labeled with CFSE (5 µM) and seeded into 48‐well plates (5 × 10^5^ cells/well) which were pre‐coated with 2 µg mL anti‐CD3 antibody (BioLegend, USA). Then, purified neutrophils from mouse tumor tissues were pretreated with PBS, LL, LI, and LLI at the same concentration of Liproxstatin‐1 (0.5 µM) without irradiation for 16 h and mixed with CD8^+^ T cell suspension at a ratio of 1:1. This was followed by the addition of 2 µg mL anti‐CD28 antibody (BioLegend) to the CD8^+^ T cell/neutrophil coculture system. After 72 h, cells were harvested and stained with fluorochrome‐conjugated anti‐mouse antibodies against CD8‐BV510, IFN‐γ‐PE, and GZMB‐PE/Cy7 for 30 min at 4 °C. After washes with PBS, cells were collected for FACS analysis.

### Animal Model

Male BALB/c mice aged 5 weeks were purchased from Gempharmatech Co., Ltd (Nanjing, China). All animal protocols were approved by the Institutional Animal Care and Use Committee of Nanjing Drum Tower Hospital (No. 2021AE01048). To establish the tumor model, MFC cells (1 × 10^6^ cells/mouse) were subcutaneously implanted in the hypochondrium. The tumor sizes and body weights of the mice were recorded every two days. Tumor volumes were calculated as the tumor length × width^2^ × 0.5.

### Multimodal Tumor Imaging

MFC tumor‐bearing BALB/c mice were injected with LLI (100 µL, 200 µg mL^−1^ Icy7) via the tail vein. Fluorescence images were acquired by the CRI maestro system (λ_ex_/λ_em_ = 740/810 nm) (CRI, USA) at pre‐determined time points after injection (0, 3, 6, 12, 24, and 48 h). At the end of the experiment, the mice were euthanized and tumors and organs (heart, liver, spleen, lung, and kidney) were collected for fluorescence imaging ex vivo. Imaging of the mice was also performed after anesthesia by inhalation of isoflurane, followed by scanning on an animal photoacoustic imaging system (PA/US Vevo LAZR‐X Fujifilm VisualSonics). After obtaining the background PA images, the mice were injected intravenously with LLI and PBS. The PA signals of the tumor site at different post‐injection time points (0, 3, 6, 12, 24, and 48 h) were measured at an excitation wavelength of 780 nm. Photothermal imaging was performed with American FLIR infrared thermal imaging equipment. PBS and LLI (100 µl, 200 µg/ml Icy7) were injected into the MFC tumor‐bearing mice via the tail vein. After 24 h, a 0.5 W cm^−2^ 808‐nm laser was used to illuminate the tumor area, and the temperature at the tumor site was recorded.

### In Vivo Antitumor Assays

When tumor volumes reached ≈120 mm^3^, the mice were randomly divided into six groups (*n* = 6 per group) and injected with 250 µL of PBS, LL, LI, and LLI via the tail vein every two days at Liproxstatin‐1 and Icy7 doses of 10 mg k^−1^g and 3.0 mg k^−1^g body weight, respectively. Twenty‐four hours after the injection, the tumor‐bearing mice were exposed to 808 nm laser irradiation for 1 min (0.5 W cm^−2^). PD‐1 antibody (200 µg mouse) (InVivoMAb anti‐mouse PD‐1) was administered intraperitoneally twice a week for a total of six doses. The tumor sizes and body weights of the mice were recorded every 2 days. When tumor volume reached ≈3000 mm^3^, mice were sacrificed, and tumors were harvested for subsequent histopathology and tumor immune microenvironment (TIME) analyses.

### Safety Analysis

To evaluate the biocompatibility and toxicity of nanoparticles in vivo, the sera of tumor‐bearing mice were obtained to evaluate liver function (AST, ALT) and renal function (CR, BUN) at the end of treatment. Meanwhile, the main organs (heart, liver, spleen, lung, and kidney) were collected from tumor‐bearing mice for H&E staining.

### Host Antitumor Immune Response

At the end of the treatment course, host anti‐tumor responses were evaluated by flow cytometry. Generally, mice were sacrificed, tumors and spleens were collected, and processed into a single‐cell suspension. To assess neutrophil ferroptosis, the suspension of cells was stained with antibodies of anti‐mouse CD45‐APC/Cy7, CD11b‐FITC, Ly6G‐PE, and CD71‐PE/Cy7. To evaluate T‐cell subpopulations, the suspension of cells was stained with antibodies of anti‐mouse CD45‐APC/Cy7, CD3‐PE/Cy7, CD4‐APC, and CD8‐BV510. To determine tumor‐associated macrophages (TAMs), the suspension of cells was stained with antibodies of anti‐mouse CD45‐APC/Cy7, CD11b‐FITC, F4/80‐APC, CD206‐PE/Cy7, and CD86‐PE. To detect mature DCs, the suspension of cells was stained with antibodies of anti‐mouse CD45‐APC/Cy7, CD11c‐PE/Cy7, CD80‐APC, and CD86‐PE. To determine PD‐L1expression, the suspension of cells was stained with antibodies of anti‐mouse CD45‐APC/Cy7, CD11b‐Percp/Cy5.5, CD11c‐PE/Cy7, and PD‐L1‐APC. To evaluate effector molecules IFN‐γ and GZMB from CD8, the suspension of cells was stained with antibodies of anti‐mouse CD45‐APC/Cy7, CD8‐BV510, IFN‐γ‐PE, and GZMB‐PE/Cy7. To analyze the immune memory, the suspension of cells from the spleen was stained with antibodies of anti‐mouse CD45‐APC/Cy7, CD8‐BV510, CD44‐FITC, and CD62L‐PE. All fluorochrome‐conjugated antibodies in this part were purchased from BioLegend.

### Histopathology Analysis

At the end of treatment, mice were sacrificed, and tumors were collected, fixed in formalin, paraffin‐embedded, and sectioned. The sections were first deparaffinized and rehydrated, followed by blocking with 10% BSA. The sections were stained with primary antibodies, followed by secondary antibodies, and imaged under optical microscopy or CLSM.

### Statistical Analysis

The data were presented as mean ± SD. Quantitative data were analyzed using Student's t‐test or one‐way analysis of variance (ANOVA). Pearson correlation analysis was performed to calculate the corresponding *R* and *p* values of the scatter plot. Origin 8.0 (Microcal Software, USA) and Prism V.7.0 (California, USA) were adopted for data analysis. ^*^ *p* < 0.05, ^**^ *p* < 0.01, ^***^ *p* < 0.001, ^****^ *p* < 0.0001, and ns, not significant. *p* < 0.05 was considered to indicate a statistically significant difference. All experiments were independently repeated at least thrice.

## Conflict of Interest

The authors declare no conflict of interest.

## Author Contributions

X.Z., W.Z., and X.W. contributed equally to this work and shared the co‐first authorship. Conceptualization: X.Z., W.Z. and X.W. Methodology: X.Z. and W.Z. Investigation: X.Z., Z.L., X.S., Q.C, Y.L., K.C., and S.A. Supervision: W.G., S.Y., S.L. Writing‐original draft: X.Z., W.Z., and X.W. Writing‐Review & edit: Y. Z., W.G., S.Y., S.L.

## Supporting information

Supporting Information

## Data Availability

The data that support the findings of this study are available from the corresponding author upon reasonable request.
