# Supplementary material for: Enhanced Photodynamic Therapy Synergizing with Inhibition of Tumor Neutrophil Ferroptosis Boosts Anti‐PD‐1 Therapy of Gastric Cancer
Source: Adv Sci (Weinh). 2024 Jan 17;11(12):2307870. doi: 10.1002/advs.202307870 (PMC10966534; doi:10.1002/advs.202307870)
Supplement: Supplementary file 1 — Supporting Information [file ADVS-11-2307870-s001.pdf]

ADVANCED SCIENCE
Open Access

Supporting Information

for *Adv. Sci.*, DOI 10.1002/advs.202307870

Enhanced Photodynamic Therapy Synergizing with Inhibition of Tumor Neutrophil Ferroptosis Boosts Anti-PD-1 Therapy of Gastric Cancer

Xudong Zhu, Wenxuan Zheng, Xingzhou Wang, Zhiyan Li, Xiaofei Shen, Qi Chen, Yanjun Lu, Kai Chen, Shichao Ai, Yun Zhu, Wenxian Guan, Shankun Yao* and Song Liu**

Supporting Information

Supplementary figures

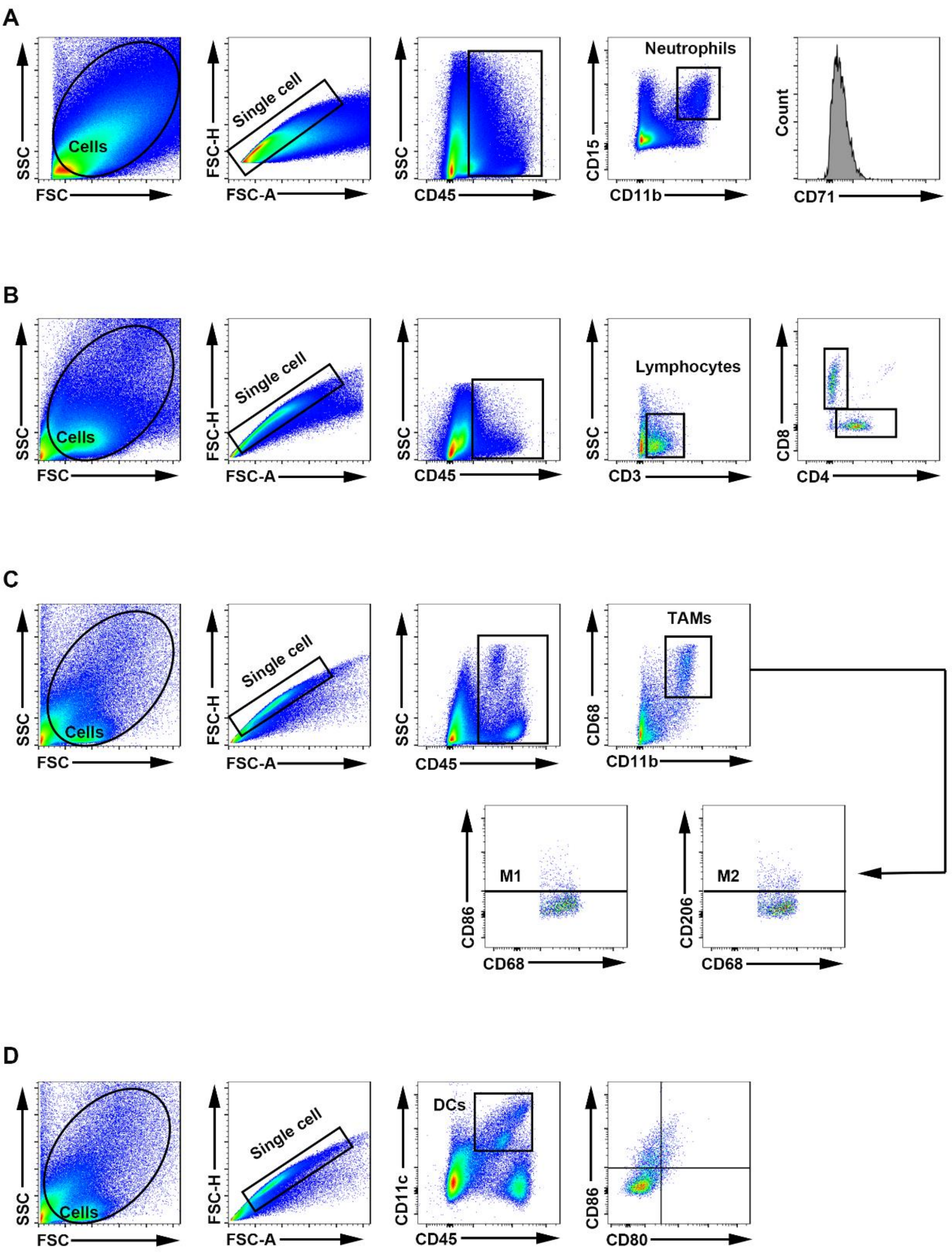

Figure S1. Gating strategies were used to investigate proportion of various immune cells in human gastric cancer samples. (A) CD71 expression was gated form CD11b^{+}CD15^{+} cells in tumors. (B) CD8^{+} T cells and CD4^{+} T cells were gated from CD3^{+} T cells in tumors. (C) M1 TAMs

and M2 TAMs were gated from CD11b^{+}CD68^{+} cells in tumors. (D) Mature DCs were gated from CD45^{+}CD11c^{+} cells in tumors.

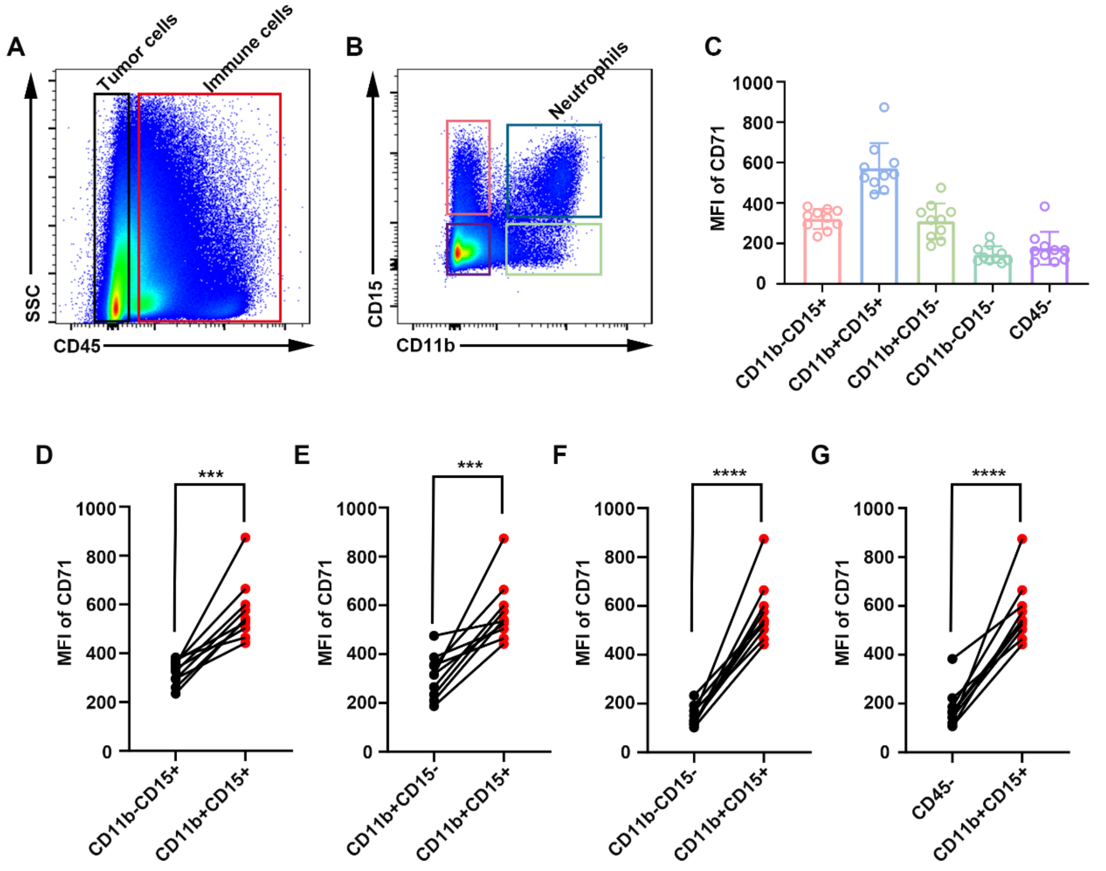

Figure S2. Ferroptosis of cell types from tumor microenvironment of gastric cancer. (A and B) Different cell populations in the tumor microenvironment gated by relevant markers. (C to G) Statistical analysis of CD71 expression on different cell populations in tumor microenvironment of gastric cancer. (Data were presented as the mean ± SD, ***p<0.001 and ****p<0.0001).

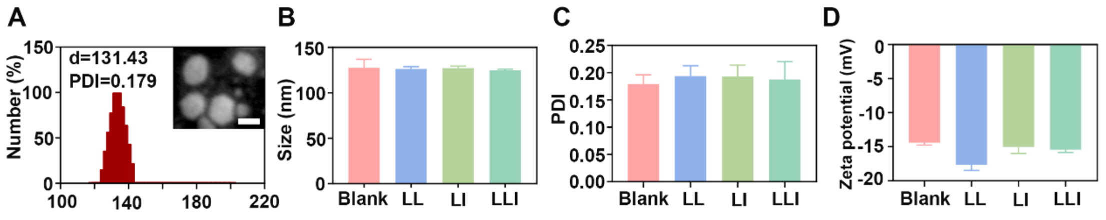

Figure S3. Size, polydispersity index (PDI), and Zeta potential of nanodrugs. (A) Size distribution and transmission electron microscope image of blank liposome. Scale bar, 100 nm (B) Size of blank liposome, LL, LI, and LLI, n=3. (C) PDI of blank liposome, LL, LI, and LLI, n=3. (D) Zeta potential of blank liposome, LL, LI, and LLI, n=3. (Data were presented as the mean ± SD).

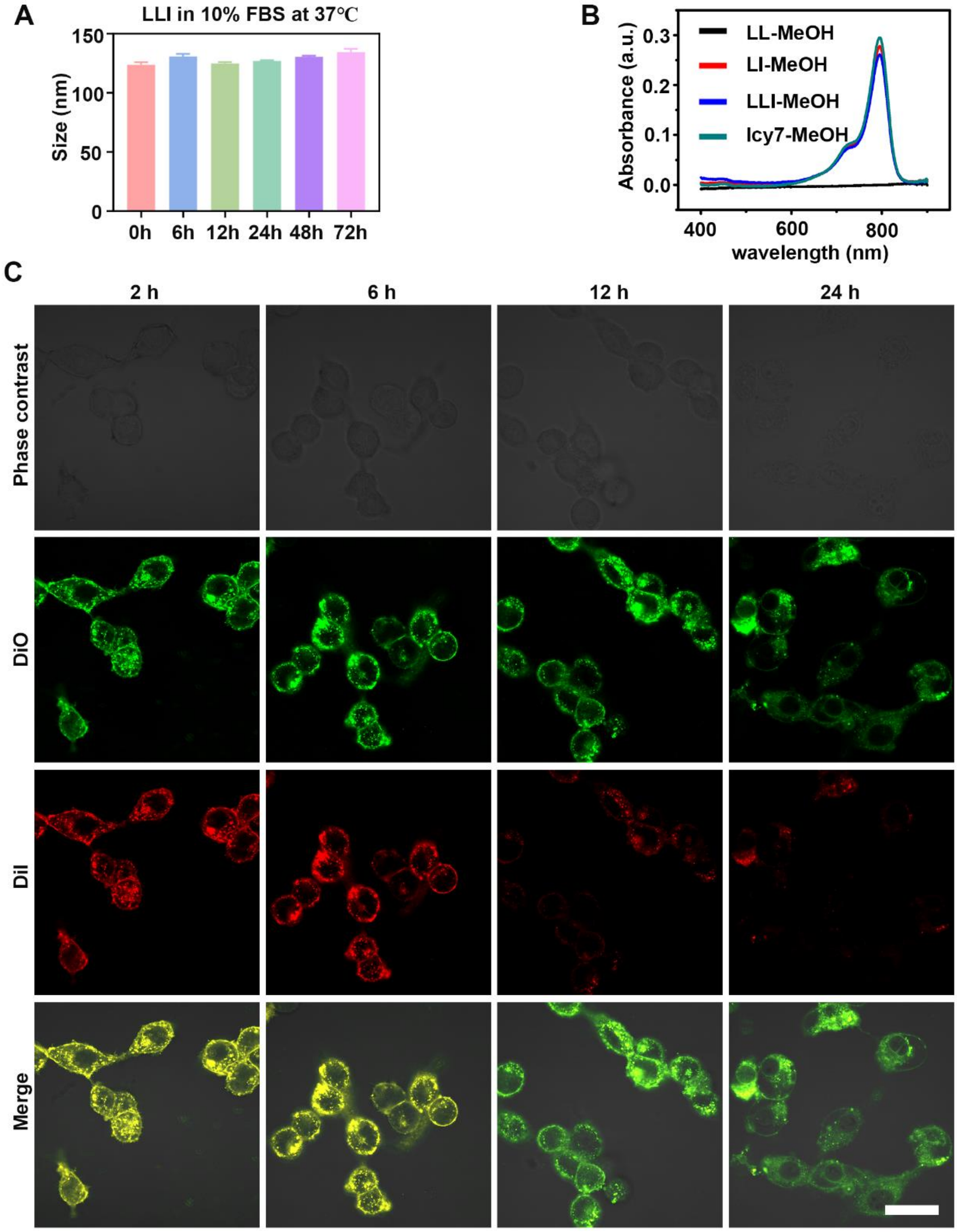

Figure S4. Stability and absorption spectra of nanodrugs. (A) Stability of LLI in phosphate buffer saline (PBS) containing 10% FBS at 37 °C, n=3. (B) Absorption spectra of LL, LI, LLI, and free Icy7 in MeOH. (C) The red fluorescence of DiI begins to fade after 12 hours, indicating the release of nanodrugs after cocultured with cells (DiO; blue), and (DiI; red), n=3. Scale bar, 50 μm. (Data were presented as the mean ± SD).

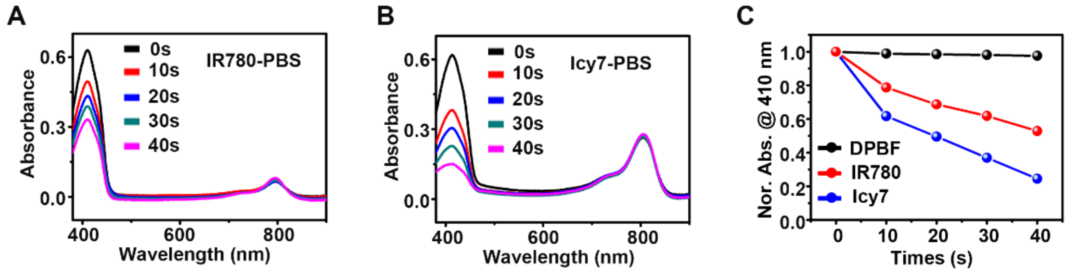

Figure S5. Production of $^1O_2$ induced by IR780 or Icy7 in PBS. (A and B) Absorption spectra of 1,3-diphenylisobenzofuran (DPBF), and DPBF degradation corresponds to the production of $^1O_2$ induced by IR780 or Icy7 in PBS under 808 nm light irradiation (0.5W/cm^2). (C) Statistical analysis of DPBF degradation.

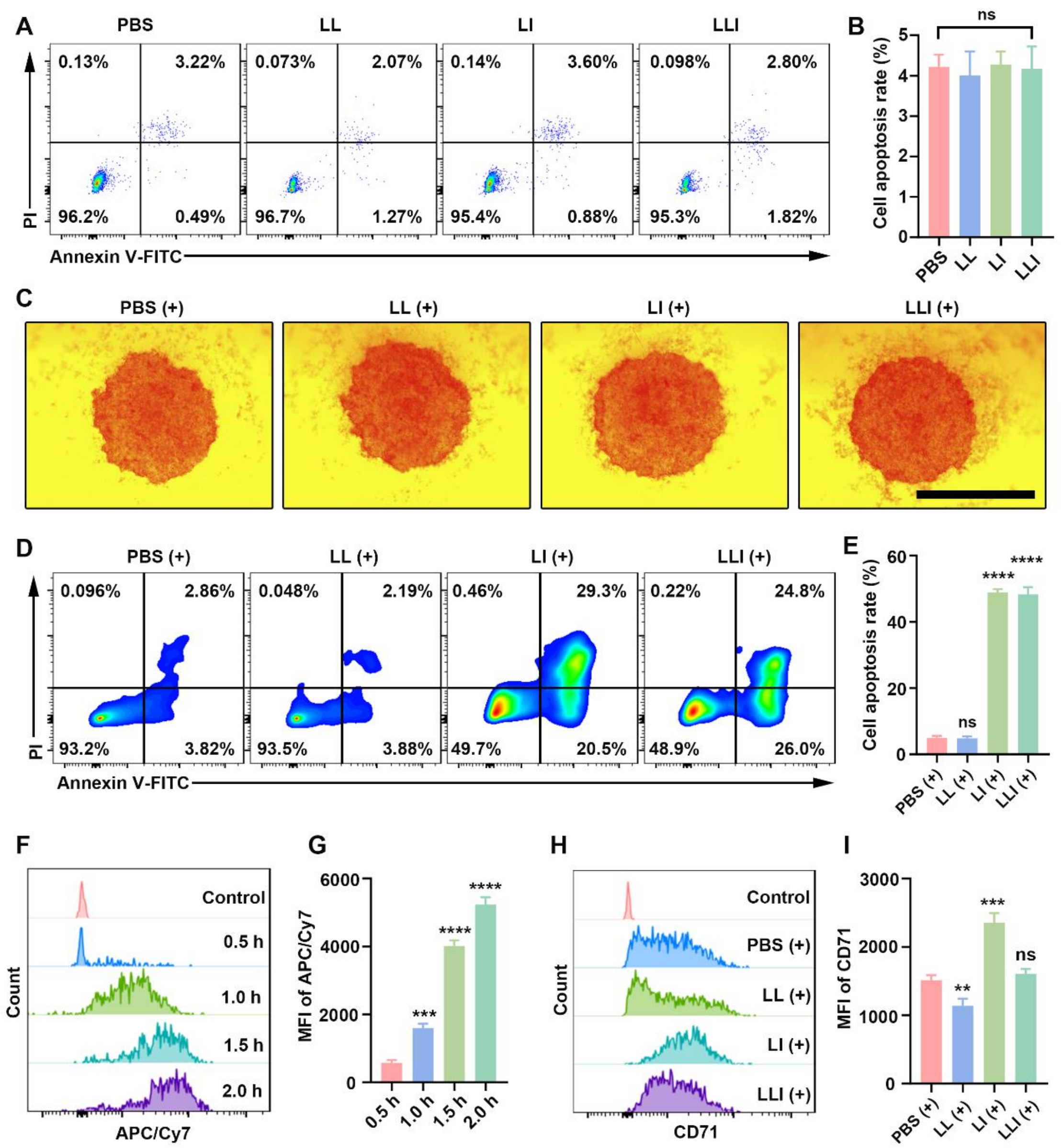

Figure S6. The cell uptake, apoptosis, and expression of CD71 in MFC cells were evaluated by 3D tumor spheroid models. (A) Apoptosis rate of MFC cells treated with PBS, LL, LI, and LLI without light irradiation, n=3. (B) Statistical analysis of apoptosis rate of MFC cells in different groups, n=3. (C) The formation of 3D tumor spheroid model. Scale bar, 500 μm. (D and E) The cell apoptosis of MFC cells was evaluated by 3D tumor spheroid models. (F and G) The uptake of nanodrugs in MFC cells was evaluated by 3D tumor spheroid models. (H and I) Expression of CD71 in MFC cells was evaluated by 3D tumor spheroid models. (Data were presented as the mean ± SD, $^{**}p$<0.01, $^{***}p$<0.001, $^{****}p$<0.0001, ns, not significant).

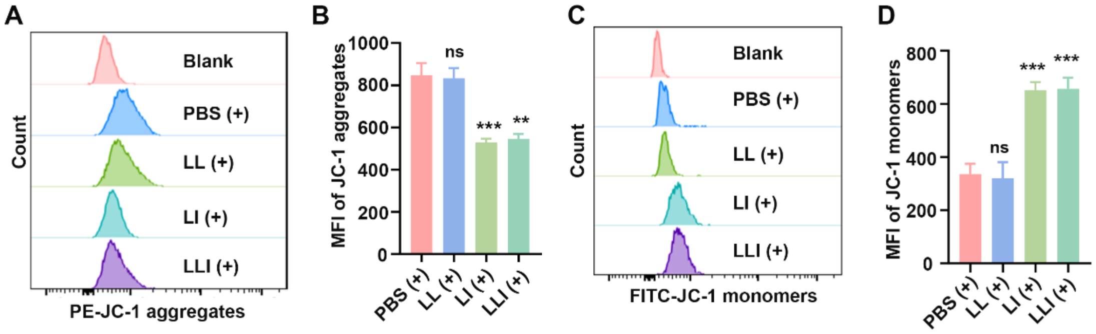

Figure S7. Mitochondria membrane potential of MFC cells determined by flow cytometry in different groups. (A) JC-1 aggregates of MFC cells determined by flow cytometry in different groups, n=3. (B) Statistical analysis of JC-1 aggregates of MFC cells in different groups, n=3. (C) JC-1 monomers of MFC cells determined by flow cytometry in different groups, n=3. (D) Statistical analysis of JC-1 monomers of MFC cells in different groups, n=3. (Data were presented as the mean ± SD, **p<0.01, ***p<0.001, and ns, not significant).

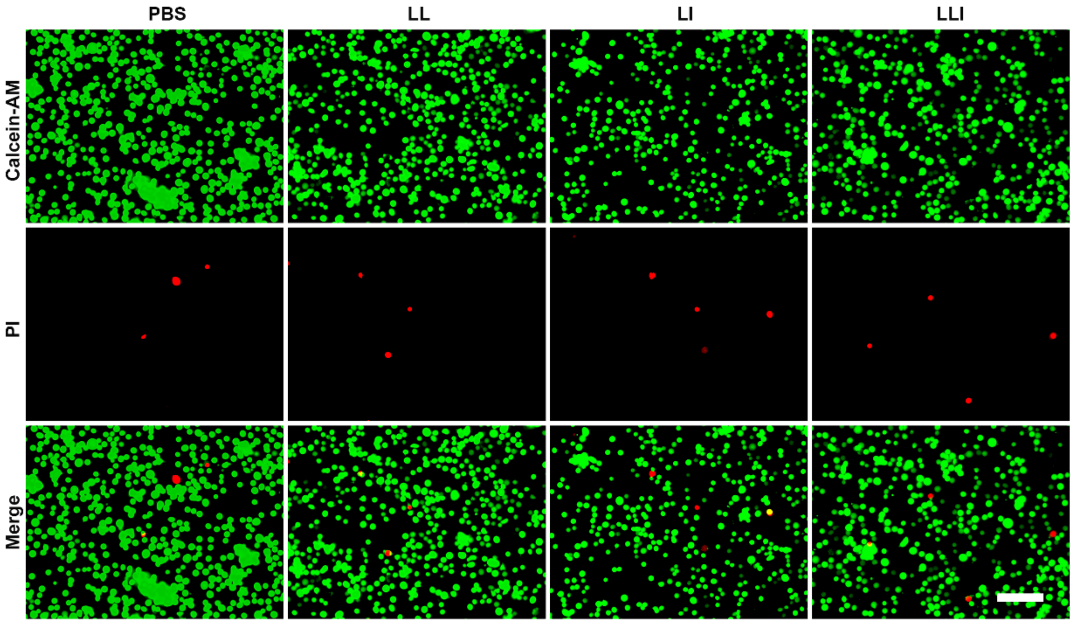

Figure S8. Live/dead cell staining of MFC cells. MFC cells incubated with PBS, LL, LI, and LLI without light irradiation (liver cells; green), and (dead cells; red), n=3. Scale bar, 100 μm.

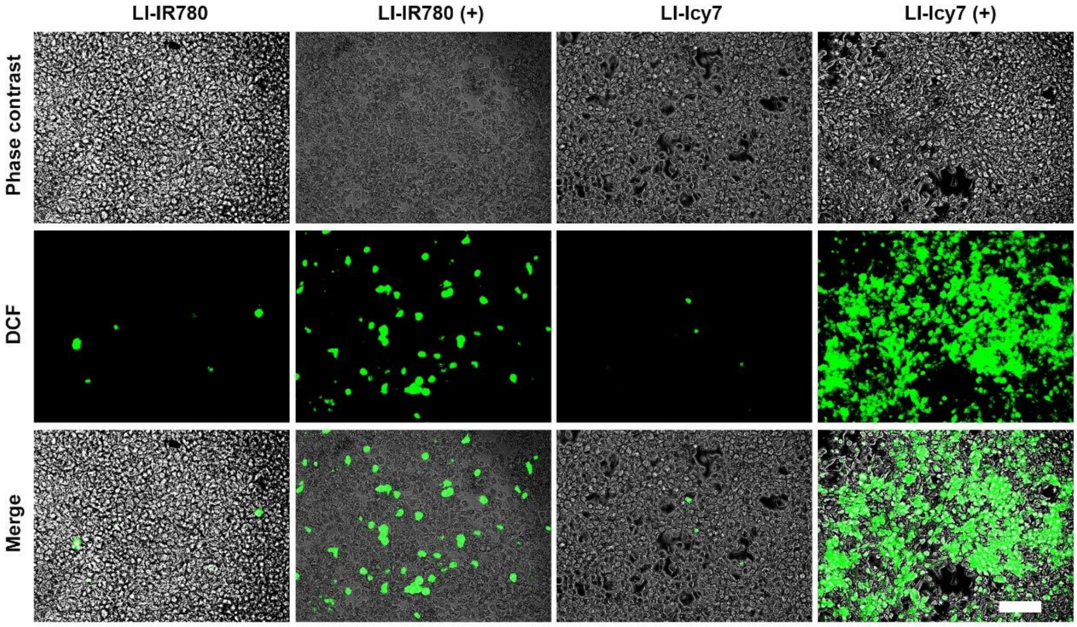

Figure S9. Fluorescence imaging of ROS production. MFC cells incubated with nanodrugs with or without light irradiation (DCF; green), n=3. Scale bar, 100 μm.

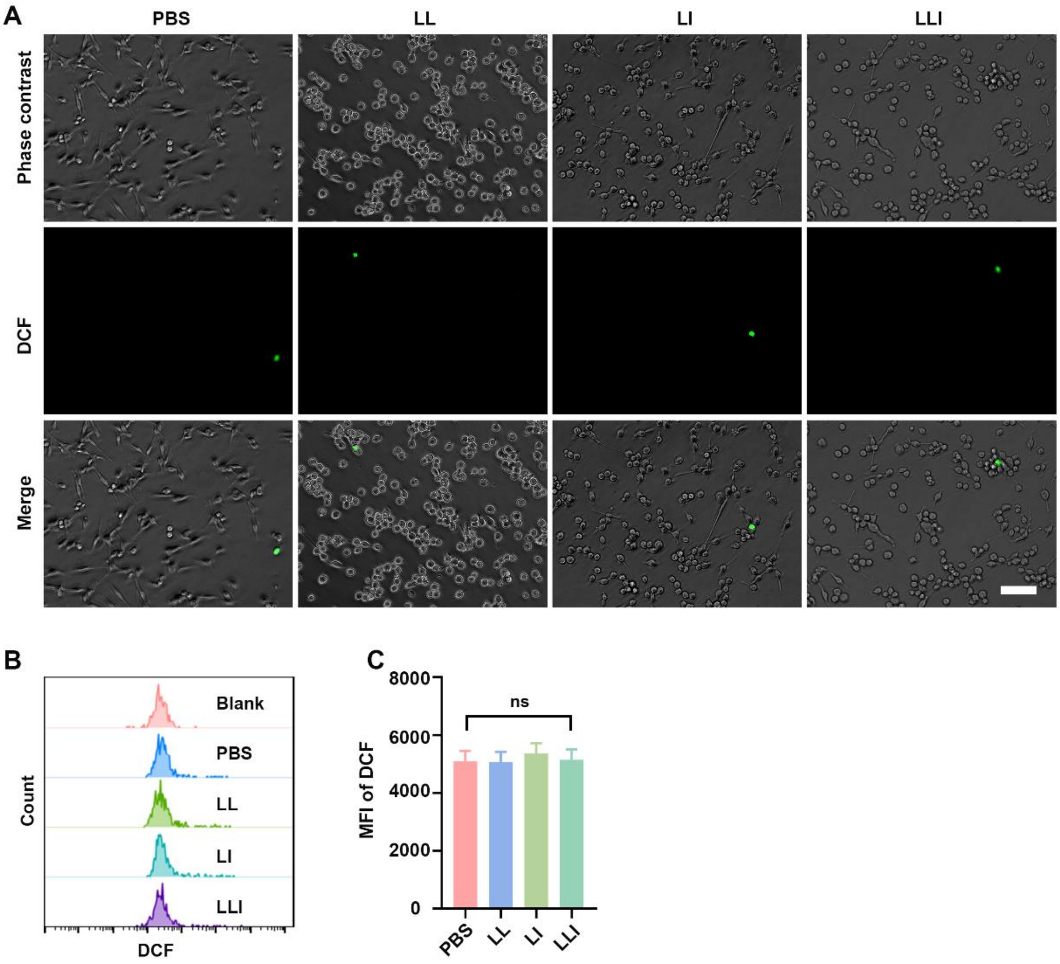

Figure S10. ROS production in MFC cells detected by confocal microscope or flow cytometry. (A) Fluorescence imaging of ROS production in MFC cells incubated with PBS, LL, LI, and LLI without light irradiation, n=3. Scale bar, 100 μm. (B and C) ROS production in MFC cells detected by flow cytometry after various treatments, n=3. (Data were presented as the mean ± SD, ns, not significant).

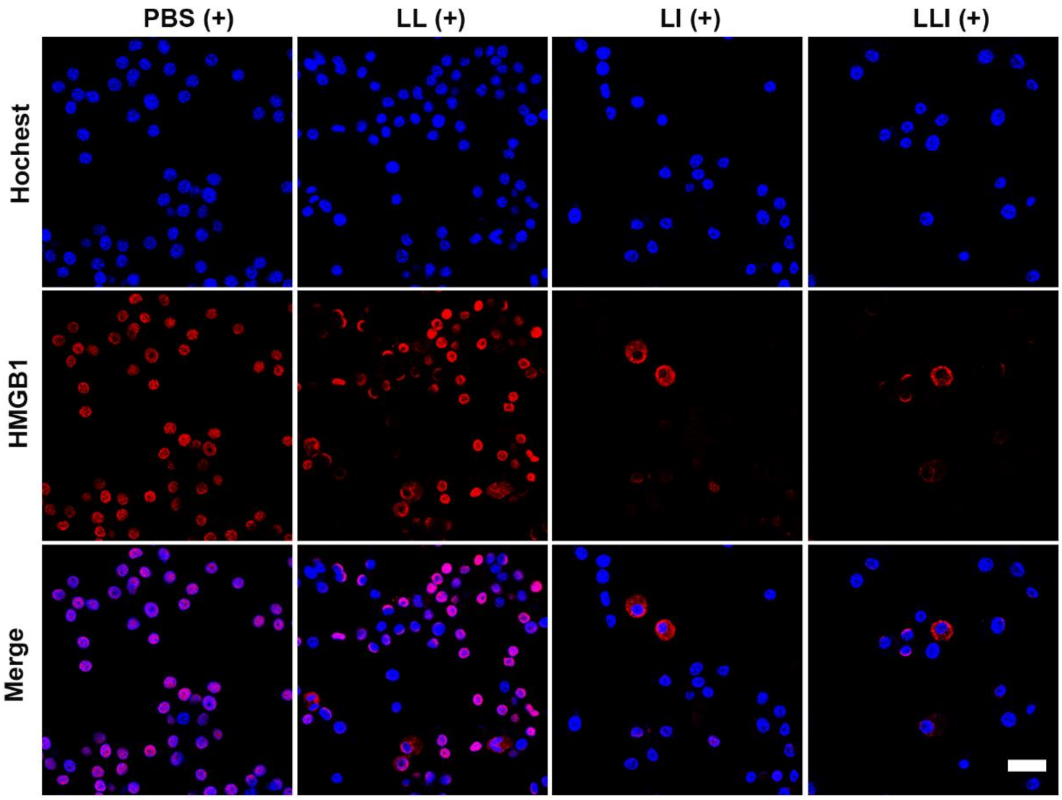

Figure S11. Nuclear HMGB1 efflux. MFC cells detected by confocal microscope in different groups (hochest; blue), and (HMGB1; red), n=3. Scale bar, 25 μm.

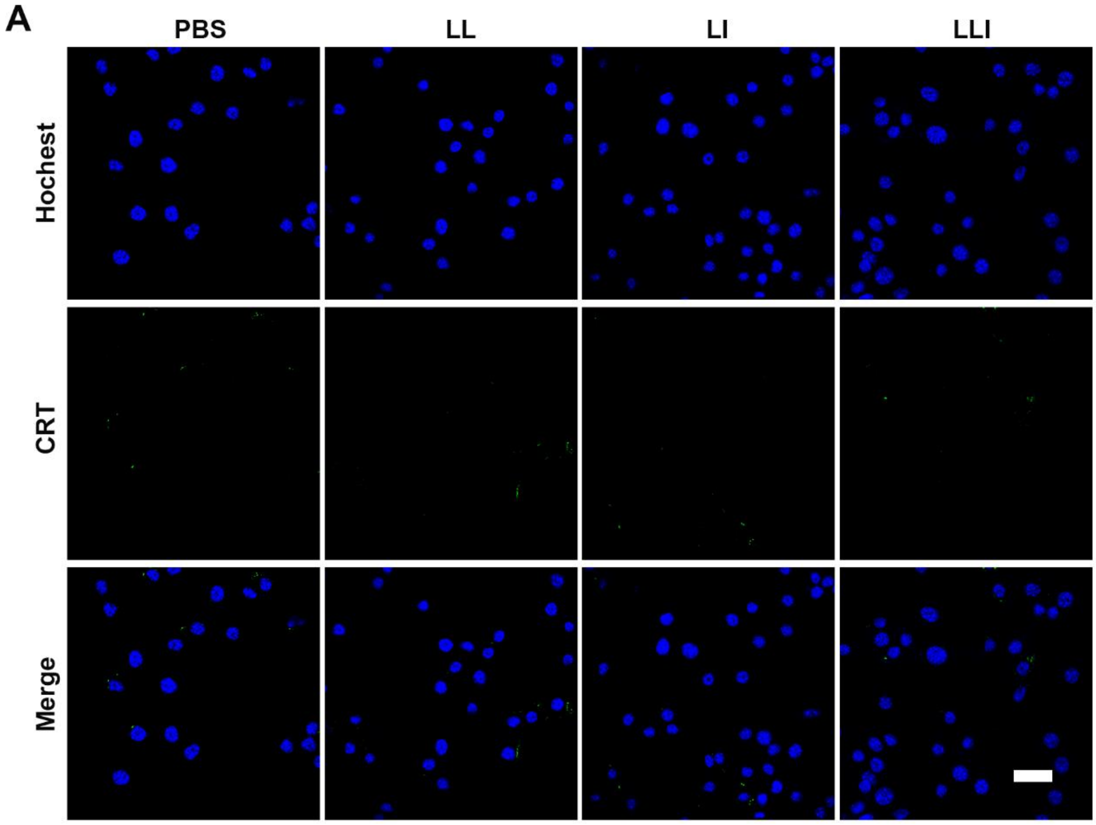
A
PBS
LL
LI
LLI
Hochest
CRT
Merge

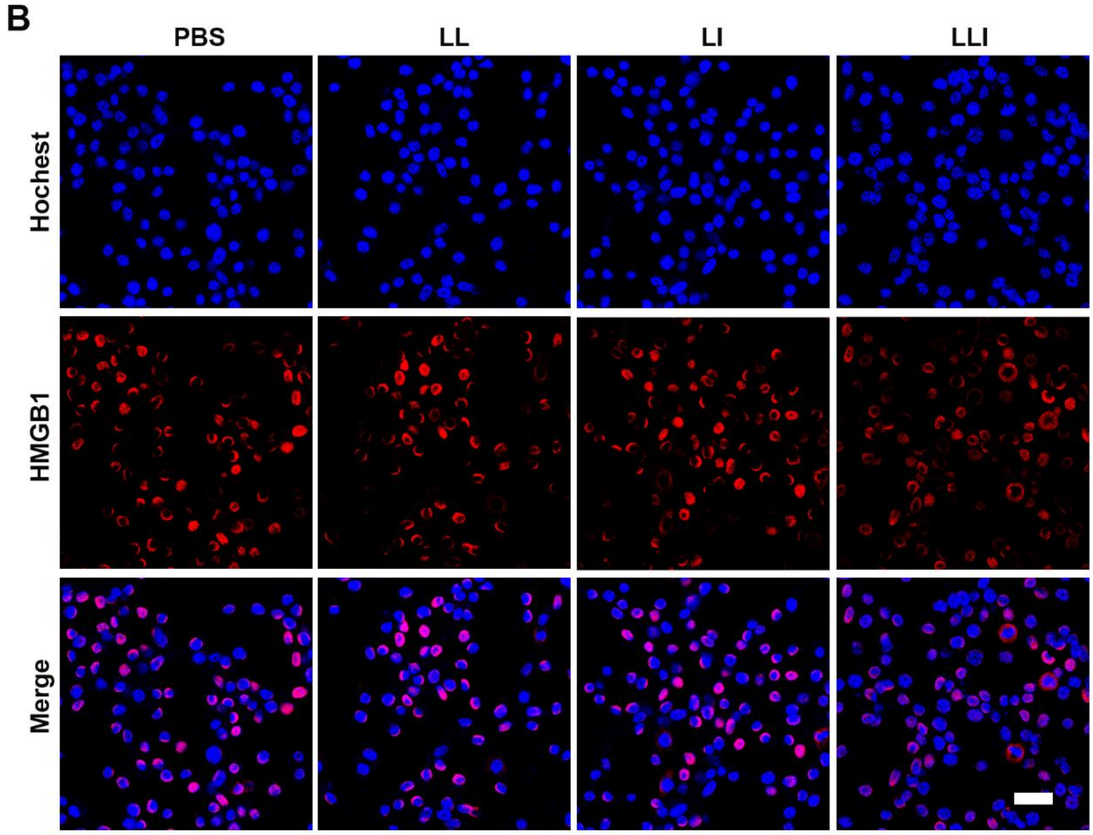
B
PBS
LL
LI
LLI
Hochest
HMGB1
Merge

Figure S12. Exposure of CRT and nuclear HMGB1 efflux detected by confocal microscope. (A) Exposure of CRT in MFC cells detected by confocal microscope in different groups (hochest; blue) and (CRT; green), n=3. Scale bar, 25 μm. (B) Nuclear HMGB1 efflux in MFC cells detected by confocal microscope in different groups (hochest; blue) and (HMGB1; red), n=3. Scale bar, 25 μm.

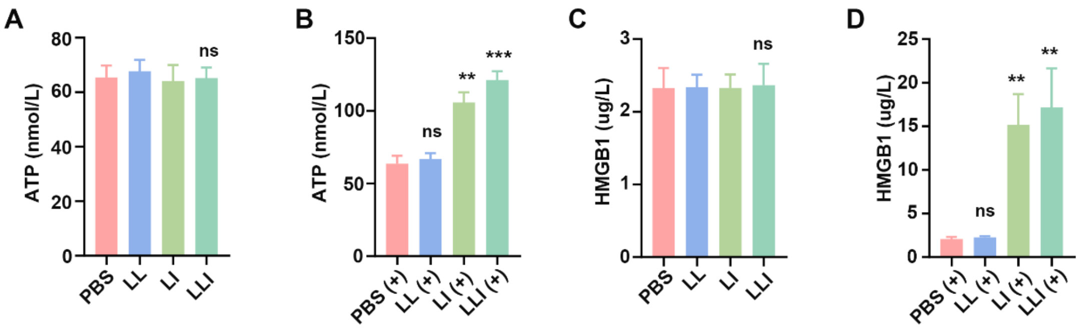

Figure S13. ATP and HMGB1 from cell supernatants were measured by using enzyme-linked immunosorbent assay (ELISA). (A and B) ATP from cell supernatants was measured by ELISA after various treatments, n=3. (C and D) HMGB1 from cell supernatants was measured by ELISA after various treatments, n=3. (Data were presented as the mean ± SD, $^{**}p<0.01$, $^{***}p<0.001$, and ns, not significant).

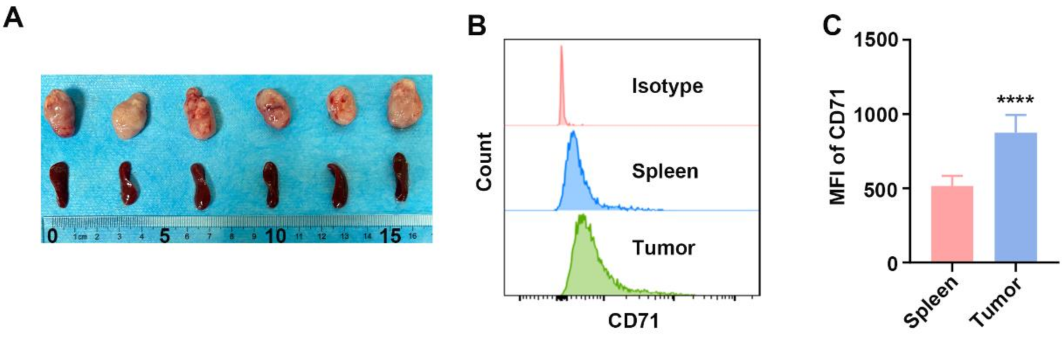

Figure S14. Ferroptosis of neutrophils in TME and spleen of MFC tumor-bearing mice. (A) Macroscopic image of tumor tissue and spleen from MFC tumor-bearing mice, n=6. (B and C) Ferroptosis of neutrophils detected by flow cytometry in TME and spleen of MFC tumor-bearing mice, n=6. (Data were presented as the mean ± SD, $^{****}p<0.0001$).

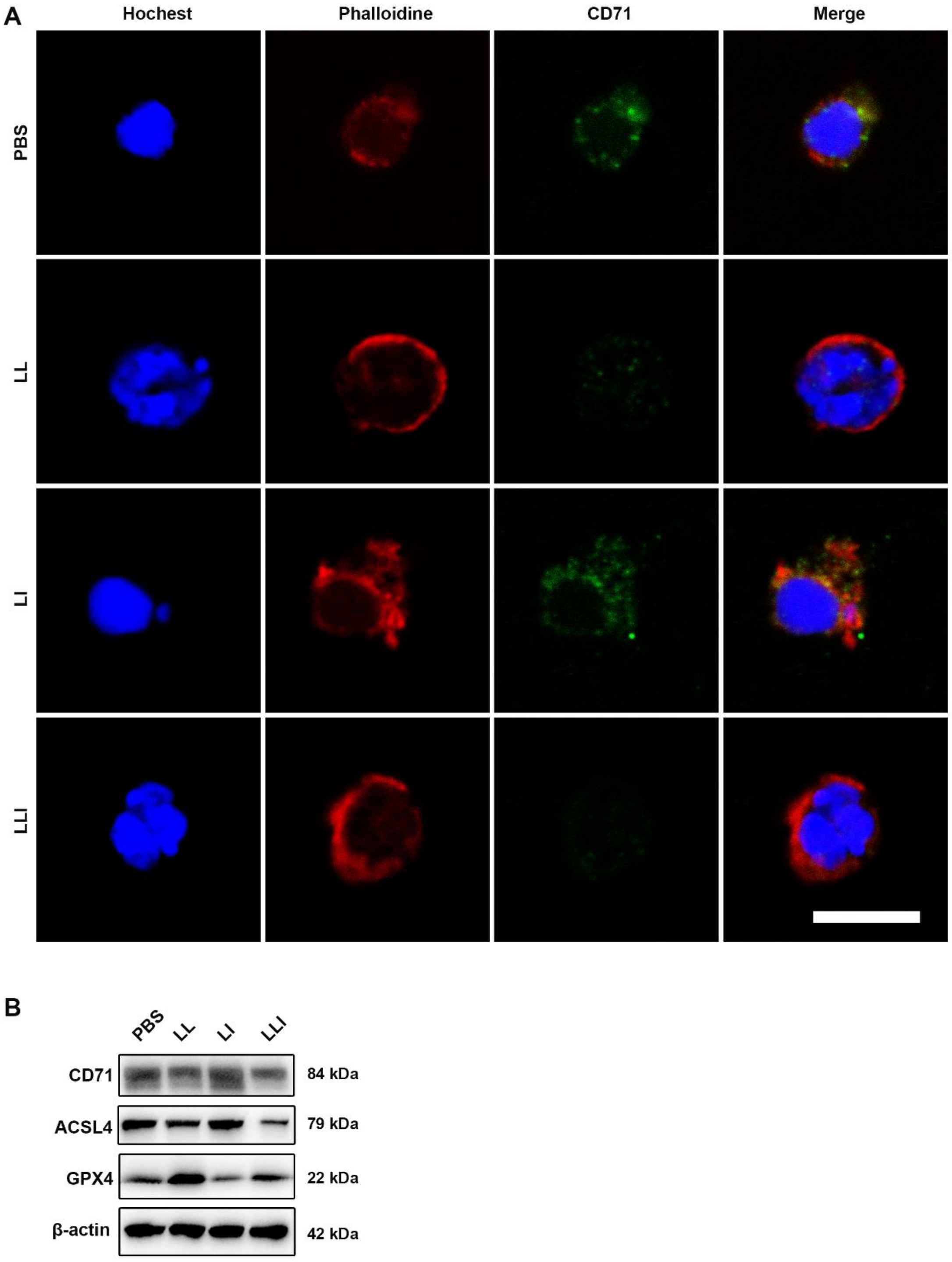

Figure S15. Relative protein expression was verified by immune fluorescence and Weston Blot. (A) CD71 of neutrophils after various treatments (hochest; blue), (CD71; green) and (phalloidine; red), n=3. Scale bar, 25 μm. (B) Expression of CD71, ACSL4 and GPX4 were observed by Weston Blot in HL60 cells.

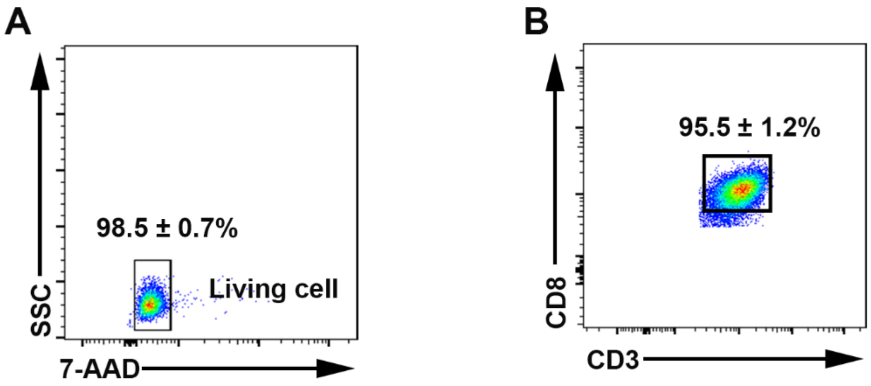

Figure S16. Viability and purity of CD8$^+$ cells from spleen of mice. (A) Viability of CD8$^+$ cells sorted by flow cytometry from spleen of mice. (B) Purity of CD8$^+$ cells sorted by flow cytometry from spleen of mice. (Data were presented as the mean ± SD).

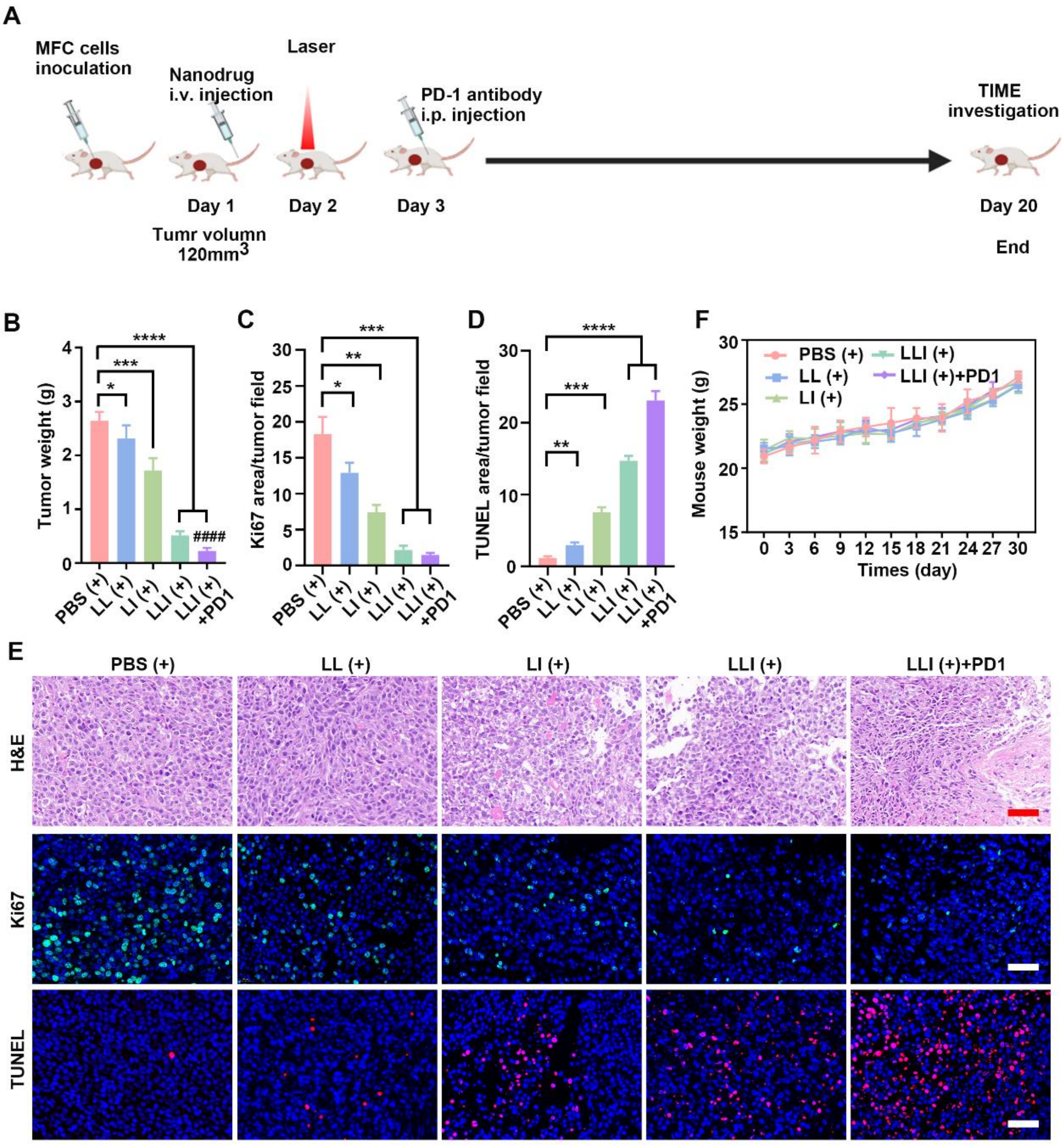

Figure S17. Synergistic anti-tumor therapy of LLI. (A) Schematic illustration of therapeutic strategy in animal model. (B) Tumor weight from mice receiving different treatments, n=6. (C) Statistical analysis of Ki67 in tumor section after different treatments. (D) Statistical analysis of TUNEL in tumor section after different treatments. (E) Representative images of tumor tissue sections showed tumor morphology (H&E staining), cell proliferation (Ki67), and apoptosis (TUNEL), n=6. Scale bar, 50 μm. (F) Changes of mice weight during various treatments, n=6. (Data were presented as the mean ± SD, **p*<0.05, ***p*<0.01, ****p*<0.001, ****/#### *p*<0.0001, #### vs. LLI).

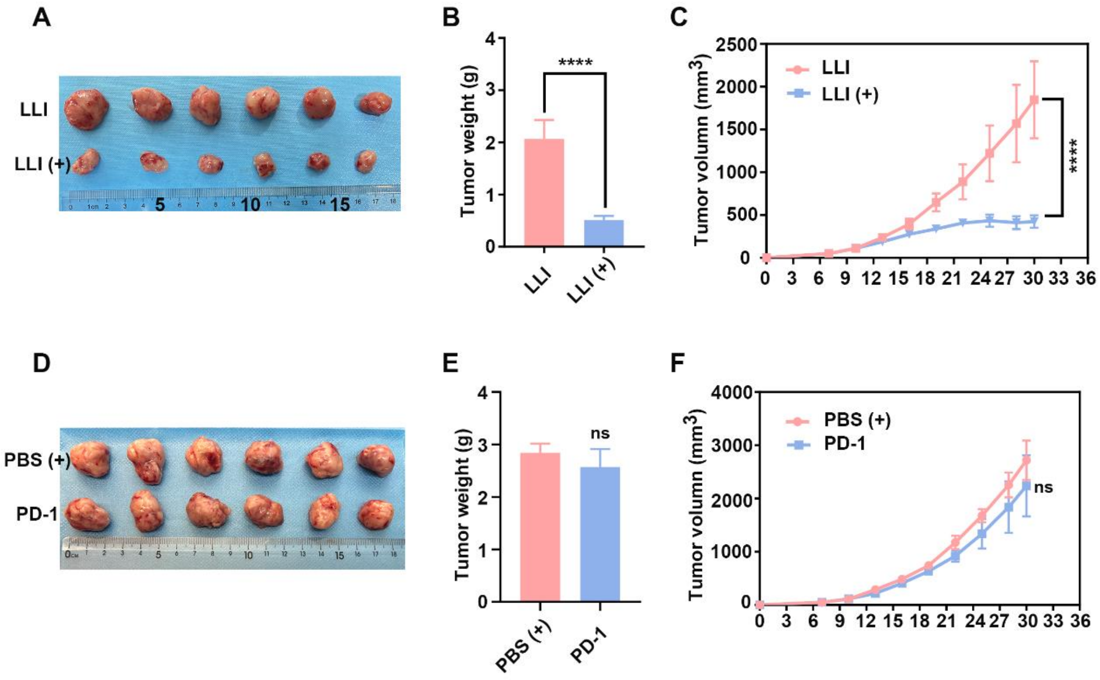

Figure S18. Tumor tissues from mice receiving different treatments. (A) Macroscopic image of tumor tissues from mice receiving LLI and LLI (+) treatments, n=6. (B) Tumor weights of mice receiving LLI and LLI (+) treatments, n=6. (C) Tumor growth curve for mice receiving LLI and LLI (+) treatments, n=6. (D) Macroscopic image of tumor tissue from mice receiving PBS and anti-PD-1 antibody treatments, n=6. (E) Tumor weights of mice receiving PBS and anti-PD-1 antibody treatments, n=6. (F) Tumor growth curve for mice receiving PBS and anti-PD-1 antibody treatments, n=6. (Data were presented as the mean ± SD, ****p<0.0001, ns, not significant).

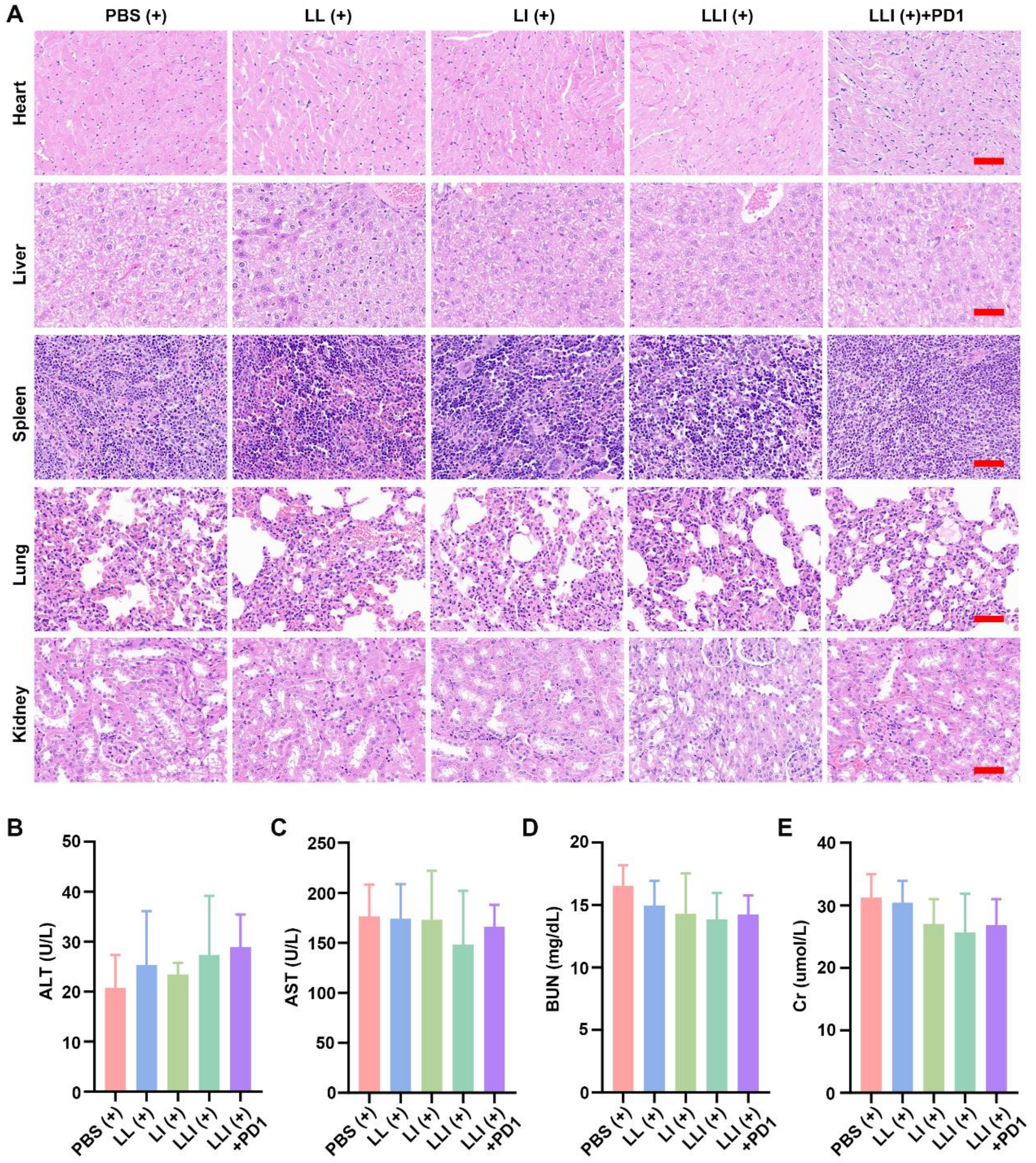

Figure S19. Biosafety evaluation of nanodrugs *in vivo*. (A) H&E staining of major organs of the treated mice, including the heart, liver, spleen, lung, and kidney, n=6. Scale bar, 50 μm. (B to E) Serum levels of liver function (ALT and AST) and renal function (Cr and BUN) were detected at the end of the treatments, n=6. (Data were presented as the mean ± SD).

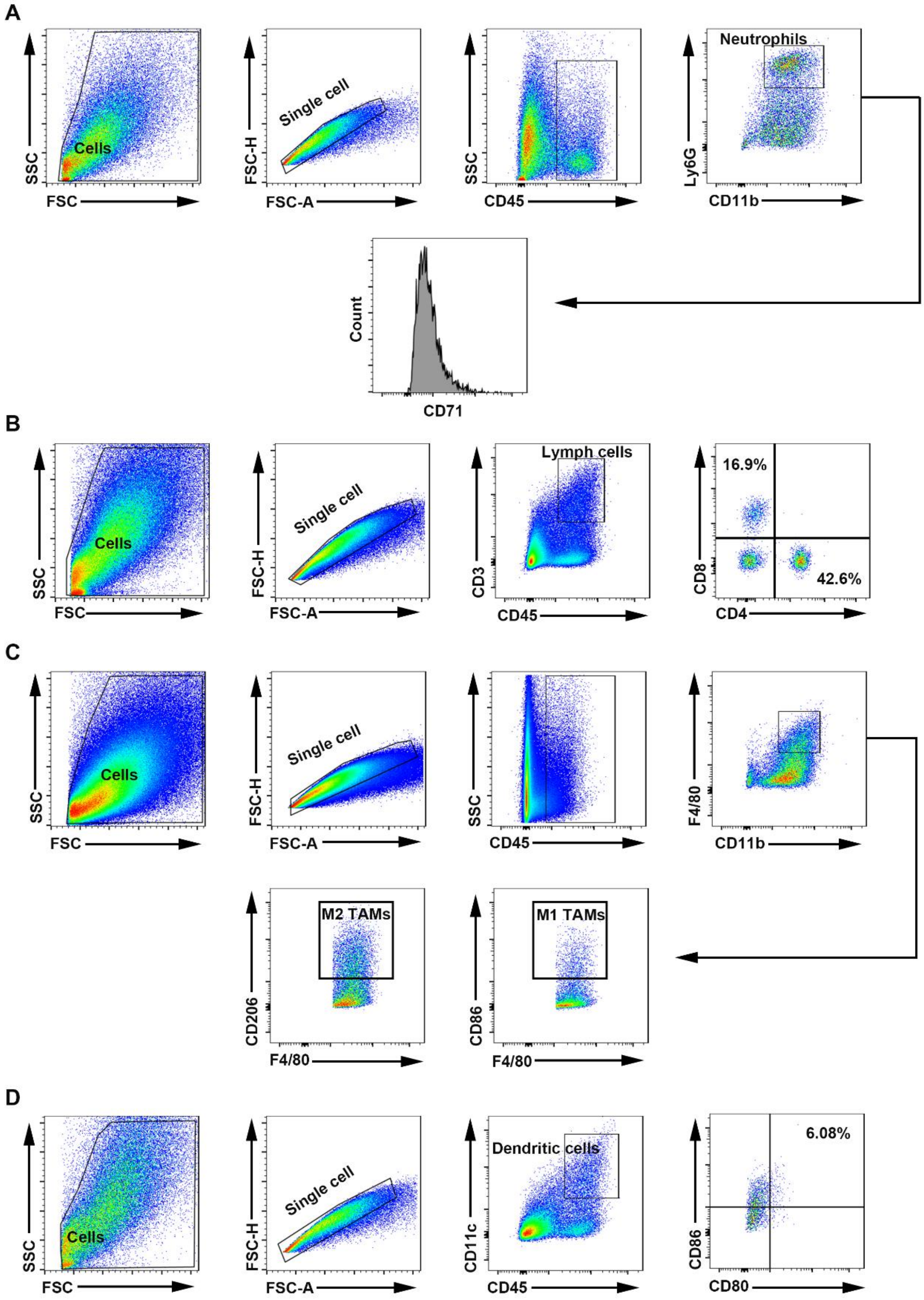

Figure S20. Gating strategies were used to investigate proportion of various immune cells in tumors of mice. (A) CD71 expression was gated form CD11b^{+}Ly6G^{+} cells in tumors. (B) CD8^{+} T cells and CD4^{+} T cells were gated from CD3^{+} T cells in tumors. (C) M1 TAMs and M2 TAMs

were gated from CD11b^{+}F4/80^{+} cells in tumors. (D) Mature DCs were gated from CD45^{+}CD11c^{+} cells in tumors.

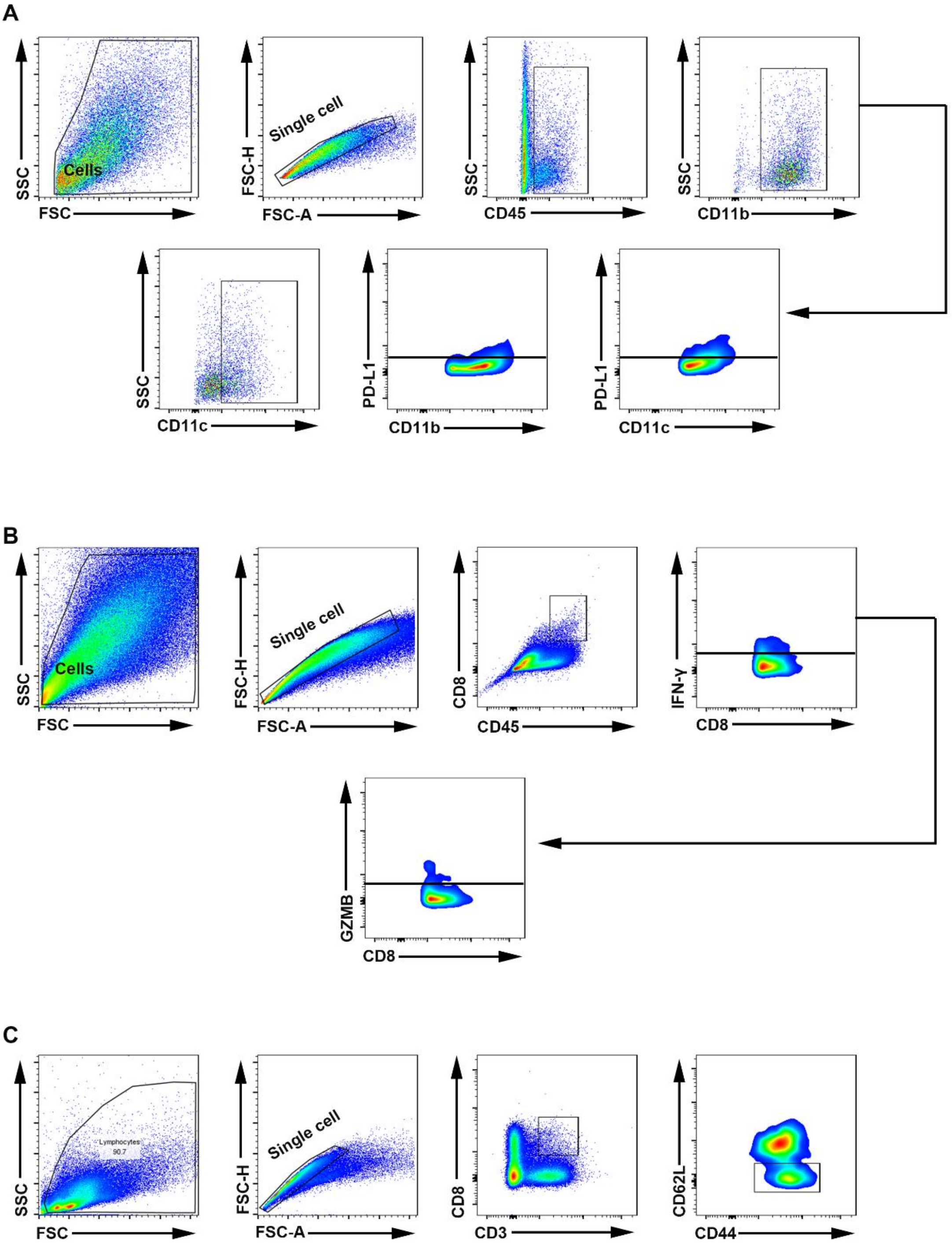

Figure S21. Gating strategies were used to investigate proportion of various immune cells in tumor or spleen of mice. (A) PD-L1 expression was gated from CD11b^{+} cells or CD11c^{+} cells.

(B) IFN-γ and GZMB production were gated from $CD45^+CD8^+$ cells in tumors. (C) Effector memory T (TEM) cells were gated from $CD45^+CD8^+$ cells in spleens.

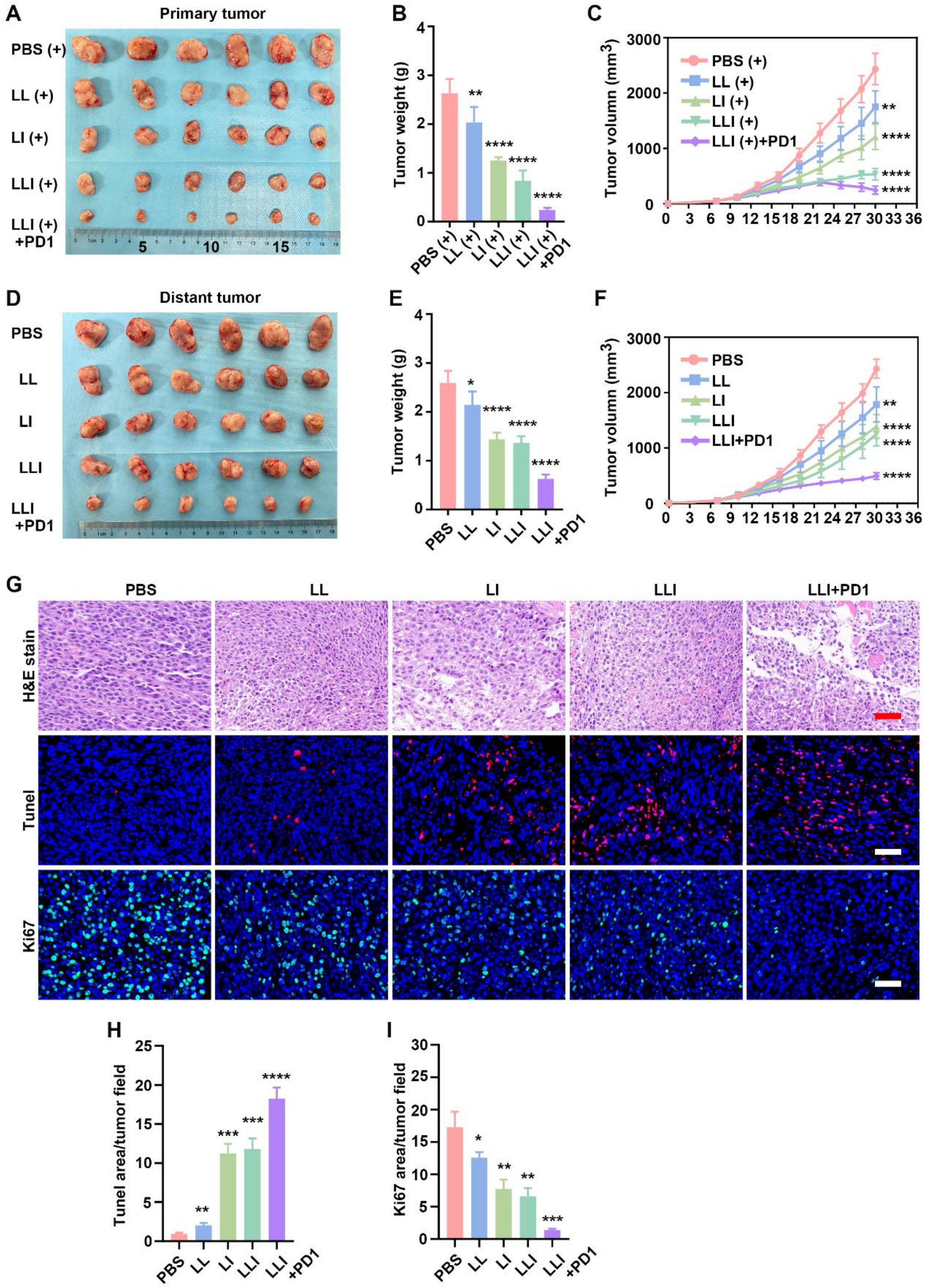

Figure S22. Nanodrugs treatments with abscopal effect. (A) Macroscopic image of primary tumor tissues from mice receiving various treatment, n=6. (B) Tumor weight of primary tumor tissues from mice receiving various treatment, n=6. (C) Tumor growth curve of primary tumor tissues from mice receiving various treatment, n=6. (D) Macroscopic image of distant tumor tissues from mice receiving various treatment, n=6. (E) Tumor weight of distant tumor tissues from mice receiving various treatment, n=6. (F) Tumor growth curve of distant tumor tissues from mice receiving various treatment, n=6. (G) Representative images of distant tumor tissue sections showed tumor morphology (H&E staining), cell proliferation (Ki67), and apoptosis (TUNEL), n=6. Scale bar, 50 μm. (H) Statistical analysis of TUNEL in distant tumor section after different treatments. (I) Statistical analysis of Ki67 in distant tumor section after different treatments. (Data were presented as the mean ± SD, *$p<0.05$, **$p<0.01$, ***$p<0.001$, and ****$p<0.0001$).

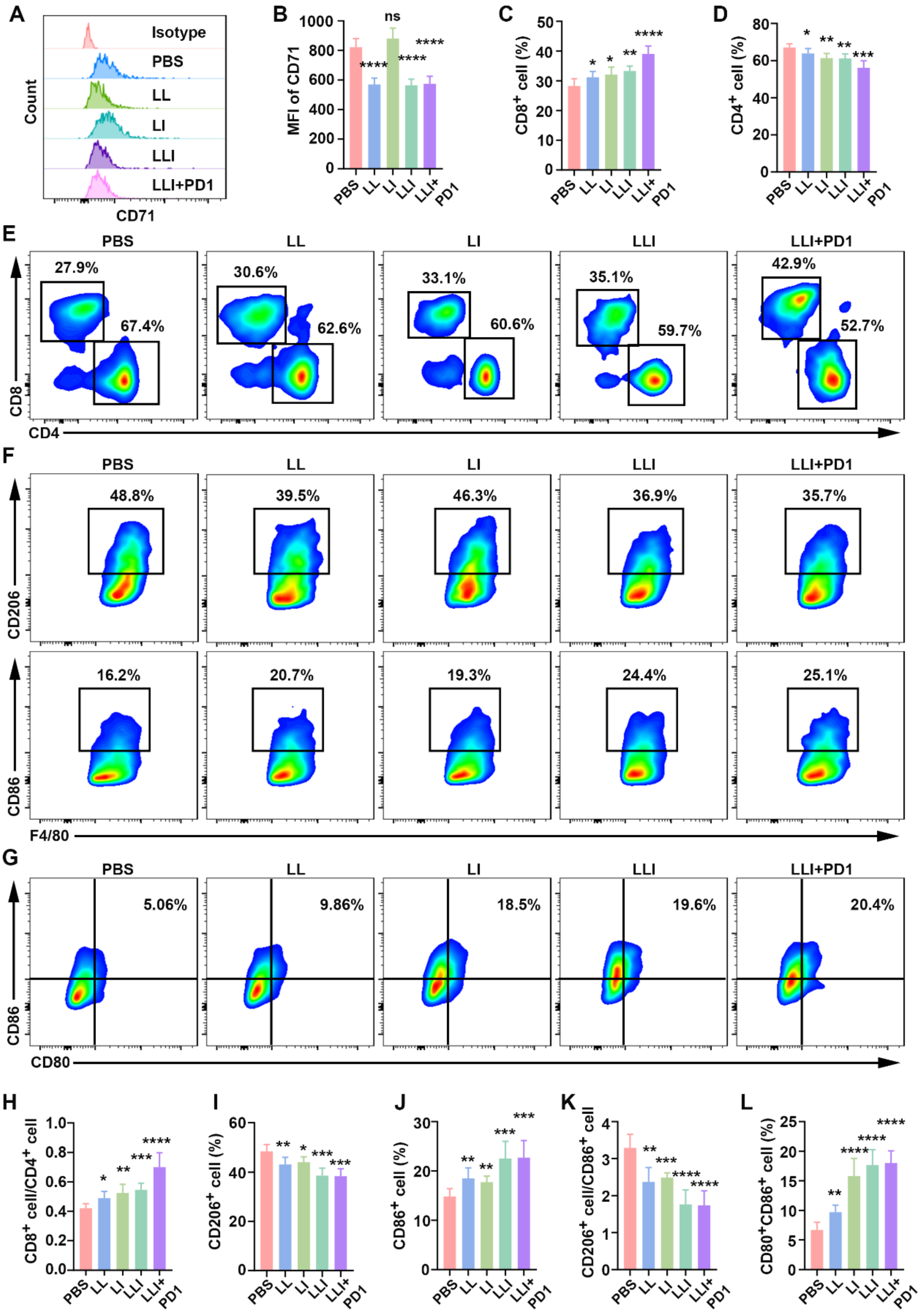

Figure S23. Nanodrugs enhanced anti-tumor immune response in distant tumors of mice. (A and B) CD71 expression of neutrophils in distant tumor tissues determined by flow cytometry after different treatments (gated on CD11b^{+}Ly6G^{+} cells), n=6. (C to E) The percentage of CD4^{+}

T cells and $CD8^+$ T cells in distant tumor tissues determined by flow cytometry after different treatments (gated on $CD3^+$ T cells), n=6. (F) The percentage of M1 and M2 TAMs in distant tumor tissues determined by flow cytometry after different treatments (gated on F4/80^+ cells), n=6. (G) The percentage of mature DCs in distant tumor tissues determined by flow cytometry after different treatments (gated on $CD11c^+$ cells), n=6. (H) Statistical analysis of the ratio of $CD8^+$ T cells to $CD4^+$ T cells in different groups, n=6. (I to K) Statistical analysis of the percentage of M1 TAMs, M2 TAMs, and the ratio of M2 TAMs to M1 TAMs in different groups, n=6. (L) Statistical analysis of mature DCs ($CD80^+CD86^+$ cells) in different groups, n=6. (Data were presented as the mean ± SD, *p<0.05, **p<0.01, ***p<0.001, and ****p<0.0001).

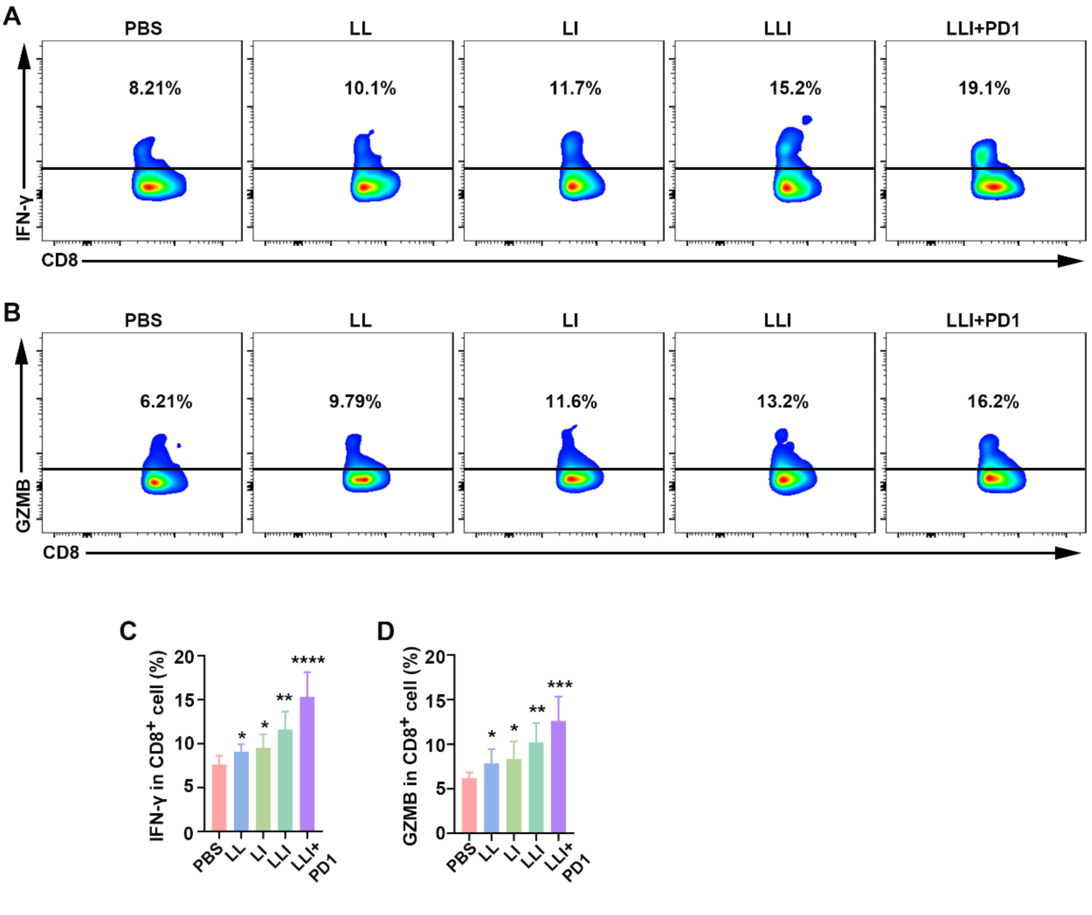

Figure S24. Nanodrugs enhanced effector molecules expression in distant tumors of mice. (A to D) Production of IFN-γ and GZMB in $CD8^+$ T cells of distant tumor determined by flow cytometry after different treatments, n=6. (Data were presented as the mean ± SD, *p<0.05, **p<0.01, ***p<0.001, and ****p<0.0001).

Synthesis of Icy7

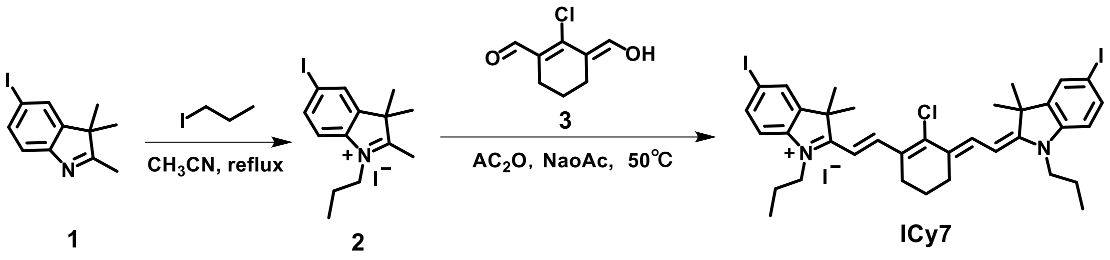

Scheme S1. Synthetic routes and chemical structures of Icy7.

Compounds 1, 2, and 3 were prepared according to the literature procedures.[1-3]

Synthesis of Icy7: Compound 2 (455 mg, 1.0 mmol) and Compound 3 (86 mg, 0.5 mmol) were dissolved in 30 mL Ac_2O solvent in a flask, and the mixture was stirred at 50 ℃ for 3 hours under argon atmosphere. After completed, the crude product was obtained by rotary distillation under reduced pressure, and was purified by silica gel column chromatography using CH_2Cl_2:CH_3OH (v/v, 98:2) as an eluent to get blue-green powder 368 mg, yield 80%. ^1H NMR (400 MHz, $CDCl_3$) δ 8.30 (d, *J* = 14.0 Hz, 2H), 7.68 (d, *J* = 8.1 Hz, 2H), 7.63 (s, 2H), 6.99 (d, *J* = 8.3 Hz, 2H), 6.23 (d, *J* = 14.0 Hz, 2H), 4.17 (t, *J* = 6.4 Hz, 4H), 2.73 (s, 4H), 1.96 (s, 2H), 1.88 (dd, *J* = 13.8, 6.9 Hz, 4H), 1.69 (s, 12H), 1.04 (t, *J* = 7.3 Hz, 6H) ppm. ^{13}C NMR (151 MHz, $CDCl_3$) δ 171.57, 150.88, 144.44, 143.14, 142.25, 137.67, 131.32, 128.38, 113.02, 101.90, 88.66, 49.22, 46.63, 28.16, 26.80, 20.89, 20.65, 11.67 ppm. HR-MS (ESI, positive mode, *m/z*): calcd. 791.11204, found 791.11041 for $[M-I]^+$.

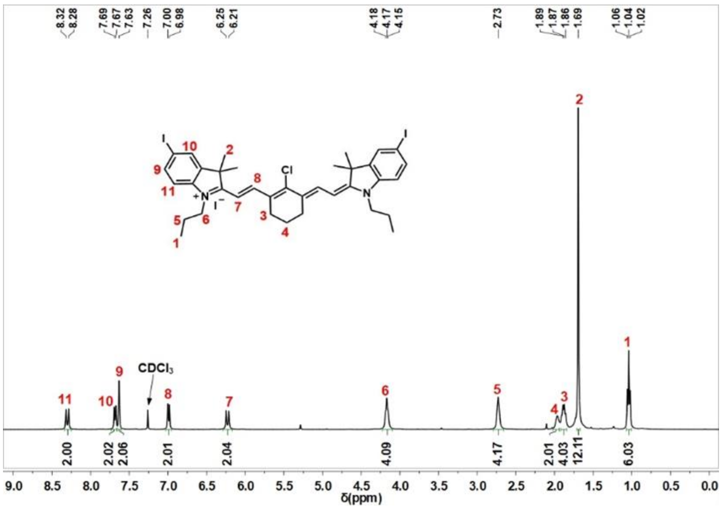

Figure S25. ^{1}H NMR spectrum of ICy7 in $CDCl_3$

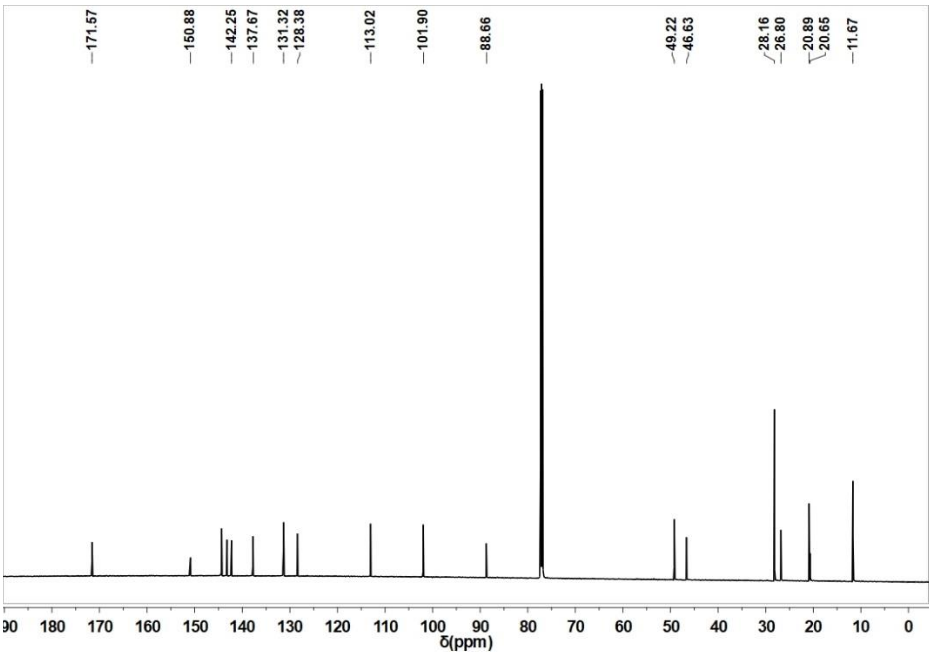

Figure S26. ^{13}C NMR spectrum of ICy7 in $CDCl_3$

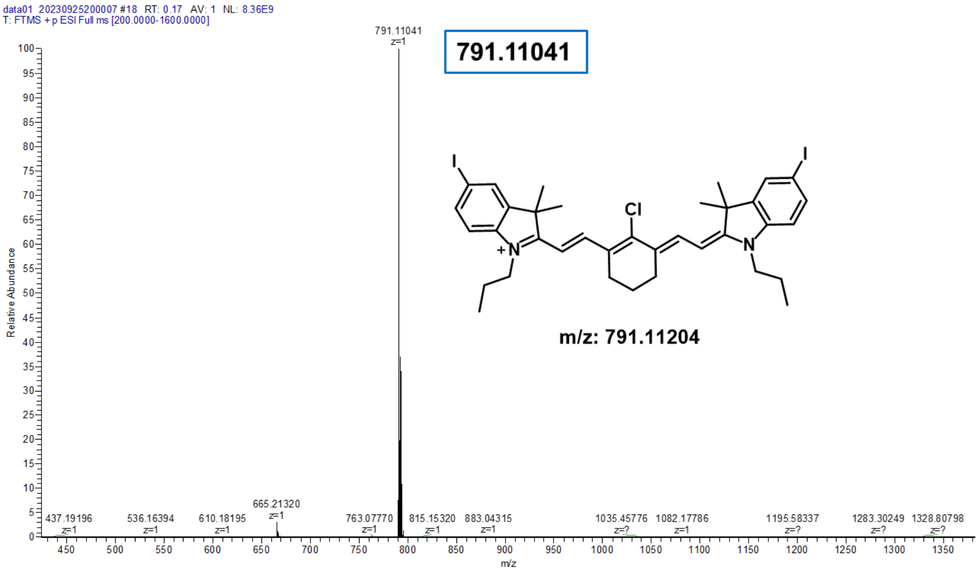

Figure S27. HR-MS spectrum of ICy7 (ESI, positive mode)

References

[1] J. Huang, K. Pu, *Chem Sci* **2021**, 12, 3379-3392.

[2] Y. Zhang, H. Liu, J. Tang, Z. Li, X. Zhou, R. Zhang, L. Chen, Y. Mao, C. Li, *ACS Appl Mater Interfaces* **2017**, 9, 17769-17776.

[3] M. Schmeisser, P. Illner, R. Puchta, A. Zahl, R. van Eldik, *Chemistry - A European Journal* **2012**, 18, 10969-10982.
